# A novel inhibitor rescues cerebellar defects in a zebrafish model of Down syndrome–associated kinase Dyrk1A overexpression

**DOI:** 10.1016/j.jbc.2021.100853

**Published:** 2021-06-04

**Authors:** Astrid Buchberger, Lena Schepergerdes, Maren Flaßhoff, Conrad Kunick, Reinhard W. Köster

**Affiliations:** 1 Division of Cellular and Molecular Neurobiology, Zoological Institute, Technische Universität Braunschweig, Braunschweig, Germany; 2 Institute of Medicinal and Pharmaceutical Chemistry, Technische Universität Braunschweig, Braunschweig, Germany; 3 Center of Pharmaceutical Engineering (PVZ), Technische Universität Braunschweig, Braunschweig, Germany

**Keywords:** zebrafish, cerebellum, Dyrk1A, Purkinje cell, neurological disease, Down syndrome, imaging, AD, Alzheimer's disease, ap, anterior–posterior, *ca8*, carbonic anhydrase 8, CLKs, Cdc2-like kinases, cpce, *ca8* promoter–derived PC-specific enhancer element, da, anterior distance, DMSO, dimethyl sulfoxide, dpf, days post fertilization, dpt, days post treatment, DS, Down syndrome, Dyrk1A, dual-specificity tyrosine phosphorylation–regulated kinase 1A, GCL, granule cell layer, ML, molecular layer, PBS, phospahte buffered saline, PCs, Purkinje cells, PFA, paraformaldehyde, SSC, saline sodium citrate, TL, torus longitudinalis

## Abstract

The highly conserved dual-specificity tyrosine phosphorylation–regulated kinase 1A (Dyrk1A) plays crucial roles during central nervous system development and homeostasis. Furthermore, its hyperactivity is considered responsible for some neurological defects in individuals with Down syndrome. We set out to establish a zebrafish model expressing human Dyrk1A that could be further used to characterize the interaction between Dyrk1A and neurological phenotypes. First, we revealed the prominent expression of *dyrk1a* homologs in cerebellar neurons in the zebrafish larval and adult brains. Overexpression of human *dyrk1a* in postmitotic cerebellar Purkinje neurons resulted in a structural misorganization of the Purkinje cells in cerebellar hemispheres and a compaction of this cell population. This impaired Purkinje cell organization was progressive, leading to an age-dependent dispersal of Purkinje neurons throughout the cerebellar molecular layer with larval swim deficits resulting in miscoordination of swimming and reduced exploratory behavior in aged adults. We also found that the structural misorganization of the larval Purkinje cell layer could be rescued by pharmacological treatment with Dyrk1A inhibitors. We further reveal the *in vivo* efficiency of a novel selective Dyrk1A inhibitor, KuFal194. These findings demonstrate that the zebrafish is a well-suited vertebrate organism to genetically model severe neurological diseases with single cell type specificity. Such models can be used to relate molecular malfunction to cellular deficits, impaired tissue formation, and organismal behavior and can also be used for pharmacological compound testing and validation.

The gene encoded by the *minibrain* locus in *Drosophila* was shown to represent a nuclear protein kinase involved in neurogenesis (1). This dual-specificity tyrosine phosphorylation–regulated kinase 1A (Dyrk1A) belongs to a highly conserved family of Dyrk kinases, and soon, a *Dyrk1A* homolog in humans was identified (2). This kinase is predominantly expressed in the central nervous system and was found to locate to the Down syndrome (DS) critical region on chromosome 21 in humans. Moreover, expression of an additional copy of *dyrk1a* in mice generated learning deficits commonly found to associate with DS (3). These findings established Dyrk1A as a crucial component of the DS critical region to elicit neurological symptoms characteristic for DS. With ongoing research, Dyrk1A was found to possess pleiotropic functions in a number of cell biological settings inside but also outside the nucleus. For example, in the nucleus, Dyrk1A phosphorylates CyclinD1 not only to mediate a cell cycle exit and induce differentiation (4, 5) but also to regulate processes of DNA repair (6, 7). In neurons, Dyrk1A also localizes to synaptosomes and is involved in regulating synapsin 1 and CaMKII function at presynapses (8). In addition, Dyrk1A was reported to bind to microtubules and regulate their dynamics in dendrites (9). In endothelial cells, Dyrk1A promotes vesicular endothelial growth factor-mediated vascularization (10). More importantly, Dyrk1A interacts with the Alzheimer's disease (AD)-causing proteins tau and amyloid precursor protein acting as priming kinase (11, 12), and this kinase has thus been implicated in the widespread appearance of AD symptoms in mouse models and humans with DS (13, 14).

Therefore, Dyrk1A has attracted a lot of attention not only by means of functional studies but also as molecular target for compound inhibitor studies to potentially treat DS. Here, a number of potent pharmacological Dyrk kinase inhibitors such as harmine, ProINDY, or Leucettine L41 have been identified and successfully tested *in vitro* and *in vivo* to rescue behavioral or cognitive defects. Although haploinsufficiency of Dyrk1A also causes severe neurological symptoms and developmental delay (15, 16, 17), these pharmacological inhibitors, when dosed well, appear to rescue Dyrk1A hyperactivity phenotypes only down to the WT level. Some common first-generation Dyrk1A inhibitors such as harmine produce significant side effects such as inhibiting monoamine oxidase (18, 19). More recent inhibitors such as ProINDY or Leucettine L41 show a better specificity and cellular uptake but still inhibit Dyrk1B, Dyrk2, or Cdc2-like kinases (CLKs) (20, 21). One of the most specific inhibitors, KuFal194 (identical with compound *5* in (22)), has been presented recently with a high Dyrk1A selectivity, yet only moderate activity was observed in cellular assays (22). KuFal194-derived inhibitors showed improved solubility, but at the expense of compromised selectivity (23).

To assess the bioactivity of Dyrk1A inhibitors, vertebrate models with Dyrk1A overexpression in a selective cell type accessible for *in vivo* imaging, behavioral analysis, and easy compound treatment protocols would be ideal. The molecularly tractable zebrafish larvae could provide such a model. It has to be pointed out, however, that owing to the partial tetraploidy of teleosts, zebrafish contains two paralogs named *dyrk1aa* and *dyrk1ab*. Nevertheless, Dyrk1A function is well conserved in zebrafish demonstrated by a loss of function mutation in *dyrk1aa* that was shown to result in social interaction impairment reminiscent of autism spectrum disorder (24). Overexpression of human or zebrafish *dyrk1a* was only performed in zebrafish primordial germ cells so far to address gonad defects in humans with DS (25).

To further address consequences of Dyrk1A hyperactivity, coexpression of human *dyrk1a* and a fluorescent protein in cells of the zebrafish nervous system would permit noninvasive *in vivo* imaging approaches to resolve phenotypes at cellular and subcellular resolution, while behavioral assays could serve to interrogate the functional consequences of Dyrk1A hyperactivity–mediated changes in neuronal physiology. If Dyrk1A function is altered only in a single cell type, behavioral phenotypes could be directly related to these cells. Moreover, as zebrafish are raised in an aqueous environment, compound administration is facilitated, while the high fecundity of zebrafish allows for treating a number of specimens simultaneously. We have therefore set out to establish such a genetic Dyrk1A hyperactivity model selectively in a single yet crucial neuronal cell type—the cerebellar Purkinje neuron in zebrafish.

## Results

### Developmental *dyrk1aa* and *dyrk1ab* widespread neural expression in larvae becomes restricted to few areas in the adult brain

The kinase Dyrk1A is highly conserved across species, but owing to a partial genome duplication in teleosts, two *dyrk1a* genes, designated as *dyrk1aa* and *dyrk1ab*, exist in zebrafish (25, 26). Both genes show an amino acid identity of 84% with respect to the entire protein and of 95% within their kinase domains. It was shown that zebrafish *dyrk1aa* is maternally expressed; its expression persists until the dome stage (25), and its developmental expression during embryogenesis has been analyzed previously (27). Yet, as the expression of the two paralogs in the maturing and adult brain has not been described in detail so far, we performed whole-mount mRNA *in situ* hybridization.

**Figure 1:**
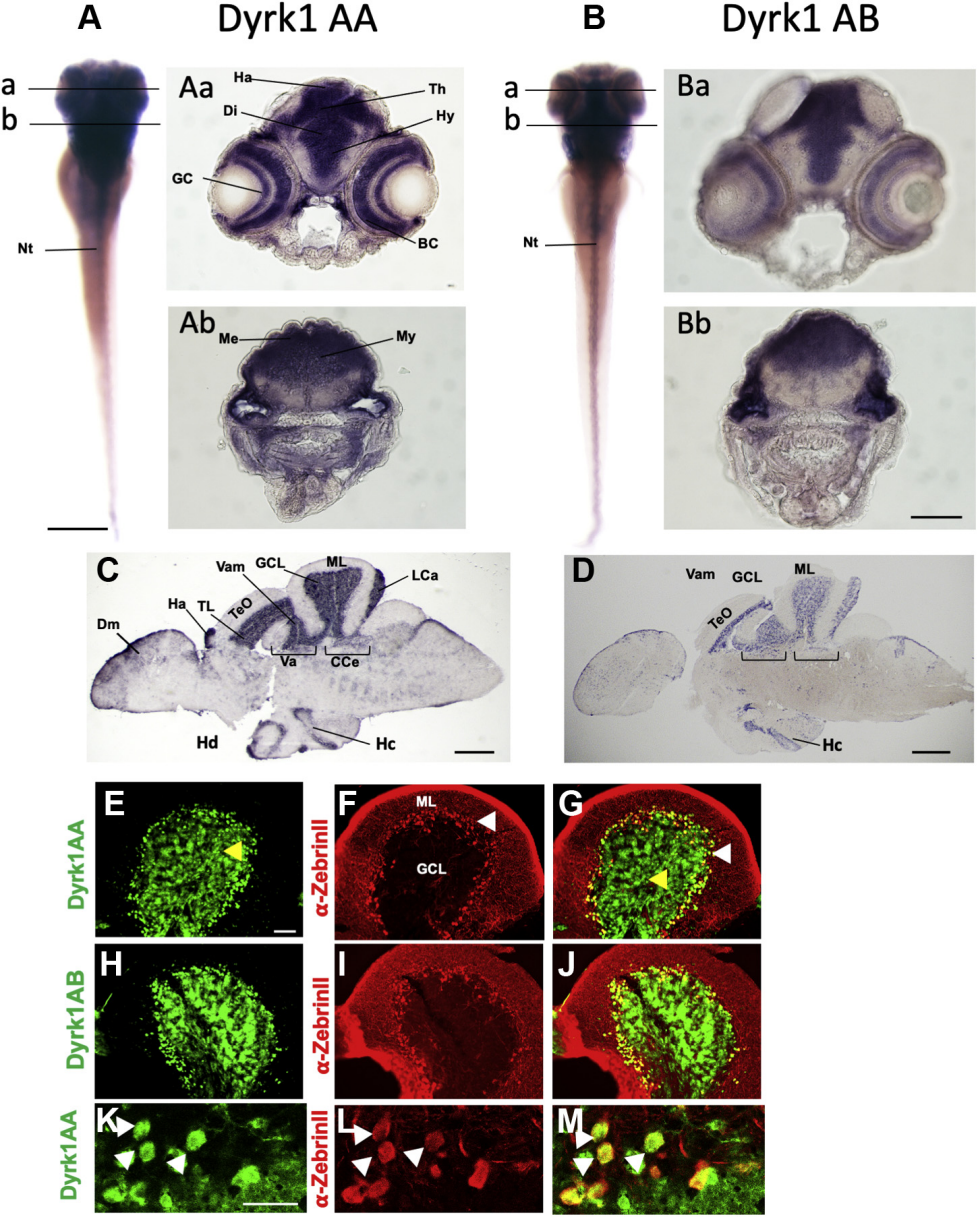
**Ubiquitous expression of *dyrk1aa* and *dyrk1ab* in the larval nervous system becomes confined to specific structures in the adult brain.** RNA *in situ* hybridization in zebrafish larvae and adult brain sections with *dyrk1aa* (*A*, *Aa*, *Ab*, *C*, and *E*–*G*) and *dyrk1ab* (*B*, *Ba*, *Bb*, *D*, and *H*–*J*) specific probes and respective sense controls are shown in Fig. S1. *dyrk1aa* and *dyrk1ab* both are expressed throughout the central nervous system of 5 dpf zebrafish larvae (*A* and *B*). Transverse sections from different planes with strong expression for both paralogs in the entire gray matter (*Aa*, *Ab*, *Ba*, and *Bb*). Chromogenic mRNA *in situ* of adult brain sagittal sections for *dyrk1aa* (*C*) and *dyrk1ab* (*D*) with intense staining in the cerebellar granule cell layer and the torus longitudinalis. Fluorescent *dyrk1aa* (*E*–*G* and *K*–*M*) and *dyrk1ab* (*H*–*J*) *in situ* with subsequent ZebrinII immunohistochemistry confirms expression in the granule cell layer below the Purkinje cell layer but also reveals expression in Purkinje cells, which was further confirmed by confocal microscopy at cellular resolution in which *dyrk1aa* expression (*K*) colocalizes with ZebrinII expression in individual Purkinje cells (*L* and *M*, *white arrows*). *dyrk1aa* (*E* and *K*), and *dyrk1ab* (*H*) fluorescent mRNA *in situ* (*green*) followed by anti-ZebrinII antibody staining (*F*, *I*, and *L*; *red*) with overlay for *dyrk1aa* (*G* and *M*) and *dyrk1ab* (*J*). *Black lines* in panels *A* and *B* indicate the plane of sections shown in panels *Aa*, *Ab*, *Ba*, and *Bb*. *Yellow* and *white arrowheads* indicate granule cells (GCs) and Purkinje cells (PCs), respectively. The scale bars indicate 200 μm (*A* and *B*), 100 μm (*Aa*, *Ab*, *Ba*, *Bb*, and *E*–*J*), 500 μm (*C* and *D*), 100 μm (*E*–*J*), and 20 μm (*K*–*M*). Bc, bipolar cells; CCe, corpus cerebelli; Di, diencephalon; Dm, medial zone of the dorsal telencephalic area; GCL: granule cell layer; Ha, habenula; Hc, central zone of the dorsal telencephalic area; Hd, dorsal zone of the periventricular hypothalamus; Hy, hypothalamus; La, lobus caudalis; Me, metencephalon; ML, molecular layer; My, myelencephalon; PCL, Purkinje cell layer; TeO, tectum opticum; Th, thalamus; TL, torus longitudinalis; Va, valvula cerebelli; Vam, medial division of valvula cerebelli.

As shown for a 5-day post fertilization (dpf) larvae, both *dyrk1a* paralogs are strongly expressed in the forebrain, midbrain, and hindbrain, the eye, and the neural tube (Fig. 1, *A* and *B*) during embryogenesis and developmental stages of brain differentiation. Sections at the diencephalon level revealed that transcripts accumulate in the habenula, thalamus, and hypothalamus and within the eye in the ganglion cell layer and in bipolar cells (Fig. 1, *Aa* and *Ba*). More caudally, at the level of the ear, expression was seen in the metencephalon including the cerebellum and the myelencephalon (Fig. 1, *Ab* and *Bb*). An analogous ubiquitous expression in the central nervous system for both genes was also observed in embryos between 1 and 4 dpf (data not shown).

In the adult brain, the expression is much more regionally restricted and particularly prominent in the cerebellum (Fig. 1, *C* and *D*). Strong *dyrk1aa* and *dyrk1ab* transcript accumulation was detected in the granule cell layer (GCL) of the corpus cerebelli, the valvula cerebelli, and the lobus caudalis, all of which represent cerebellar structures. In addition, expression was found in the torus longitudinalis (TL) below the tectum opticum, which is considered a cerebellum-like structure in teleosts (28, 29). Weaker and more diffuse expression was observed in the medial zone of dorsal telencephalic area, in the dorsal zone of the periventricular hypothalamus, and the central zone of the dorsal telencephalic area. From the chromogenic staining (Fig. 1, *C* and *D*), expression of *dyrk1aa* and *dyrk1ab* in cerebellar Purkinje cells (PCs) could not be deduced. Therefore, FISHs were performed in combination with immunohistochemistry using the PC-specific ZebrinII antibody (30). These stainings clearly revealed that both *dyrk1a* paralogs (Fig. 1, *E* and *H*) are expressed in ZebrinII-positive cerebellar PCs (Fig. 1, *F* and *I* and merged images Fig. 1, *G* and *J*). Laser confocal scanning microscopy at higher magnification with cellular resolution of *dyrk1aa*-expressing cells (Fig. 1*K*) in the ZebrinII-expressing PC layer (Fig. 1*L*) indeed confirmed coexpression of *dyrk1aa* and ZebrinII in individual PCs (Fig. 1*M*, white arrows). The same results were obtained in double fluorescence RNA *in situ* hybridizations with a PC-specific *parvalbumin7* probe (data not shown).

Taken together, *dyrk1aa* and *dyrk1ab* display a similar expression pattern in the brain of embryos, larvae, and the adults. Although during early development the expression throughout the nervous system is ubiquitously distributed in the entire gray matter, the expression in the adult brain becomes confined to specific regions and is particularly prominent in GCs and PCs of the cerebellum and the cerebellum-like TL.

### Overexpression of human Dyrk1A in cerebellar PCs of stable transgenic zebrafish

DS causes mental disability with neuromorphological, synaptic, and cognitive alterations (31, 32, 33, 34). It has been proposed that Dyrk1A, which is located within the genomic DS critical region on human chromosome 21, is implicated in the cognitive impairments seen in DS (35, 36, 37). So far, transgenic bacterial artificial chromosome (BAC) and yeast artificial chromosome (YAC) mouse models were used to evaluate the role of Dyrk1A (36, 38). In these models, the gene dosage is increased in many cell types, in which *dyrk1a* is endogenously expressed.

Based on the finding that *dyrk1a* expression in adult zebrafish is largely confined to the cerebellum, we decided to increase the dose of Dyrk1A in a single cell type. This will allow to unravel cell autonomous effects of Dyrk1A hyperactivity and to attribute physiological and behavioral consequences to a defined cell population. We targeted PCs as these represent the principal and sole output neurons of the cerebellar cortex. In addition, the human *dyrk1a* homolog was chosen to allow for pharmacological rescue approaches affecting the human homolog.

**Figure 2:**
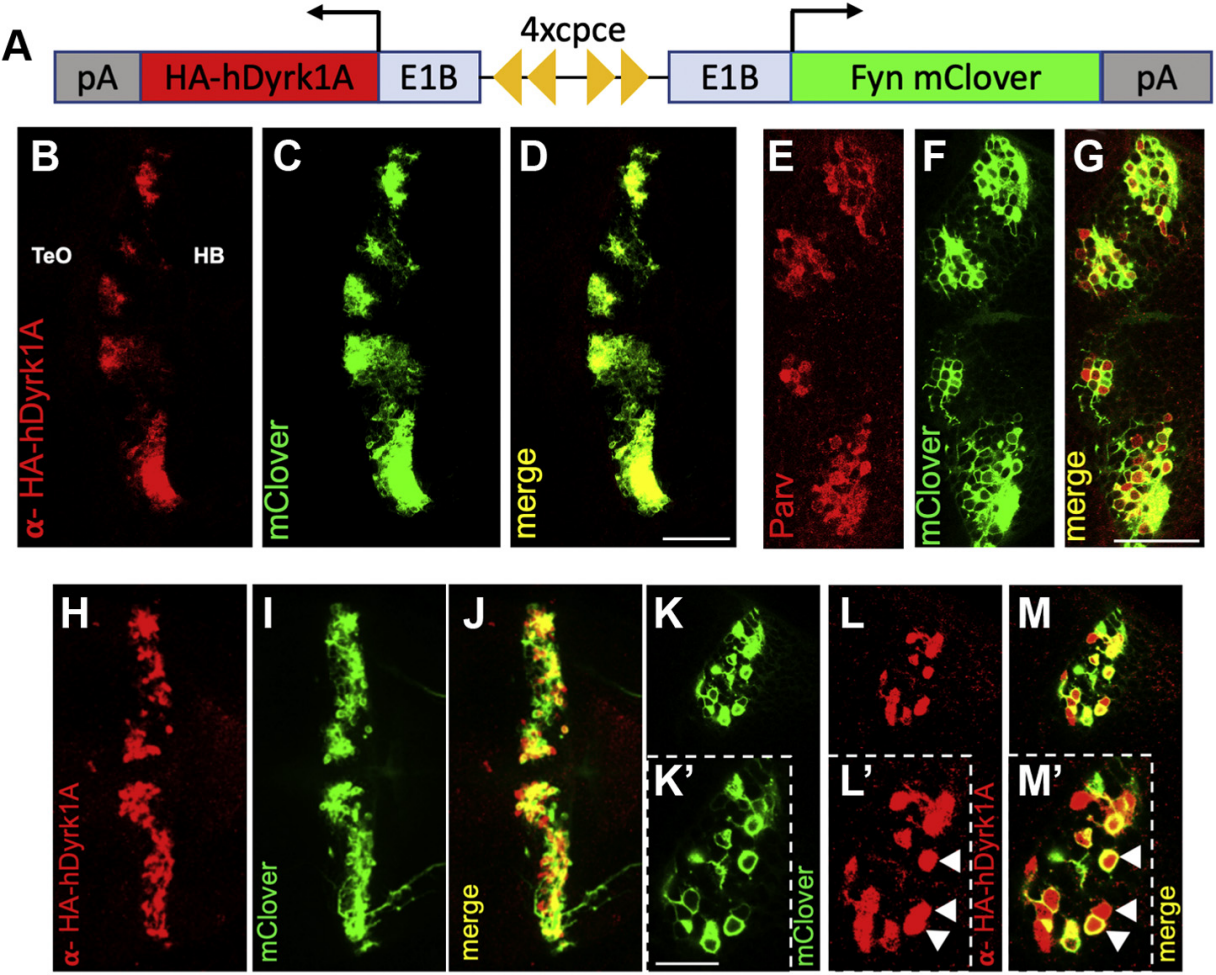
**Purkinje cell–specific expression of human Dyrk1A in zebrafish.***A*, schematic presentation of the bidirectional construct for the generation of a stable transgenic line driving HA-tagged hDyrk1A expression in Purkinje cells. Two *ca8* promoter-derived PC-specific enhancer element (cpce) dimers in opposite orientation (*yellow triangles*) are linked to E1B basal promoters on both sides directing the expression of mClover (right cistron) and HA-hDyrk1A (left cistron) to Purkinje cells. The construct is not drawn to scale. *B*–*M*, dorsal views (anterior to the *left*) onto the left and right cerebellar PC hemispheres (*B*) recorded by confocal microscopy. Transient transgenic 4 dpf larvae show expression of hDyrk1A, visualized by αHA antibody staining, and (*C*) FynmClover fluorescence in Purkinje cells. *D*, the merged image reveals coexpression of both proteins. Colocalization of the PC-specific αParvalbum antibody staining (*E*) and FynmClover (*F*) in the merged image (*G*) indicate that the hDyrk1A expression is confined to cerebellar PCs. *H*–*M*, whole-mount immunostaining on F2 larvae of a stable *Tg(ca8-hDryk1A-mClover)* line. *H*, maximal brightness projections of confocal microscopy image stack recordings after αHA antibody staining visualize hDyrk1A expression in the cerebellum. *I*, the corresponding mClover fluorescence labels PCs, whereas the merged image (*J*) demonstrates reliable coexpression of both proteins. Images at higher magnification display membrane-tagged mClover (*K* and *K’*) and nuclear/cytoplasmic hDyrk1A (*L* and *L’*) colocalized within the same cells (*M* and *M’*). *White arrowheads* indicate Purkinje cells. The scale bar represents 50 μm (*B*–*M*) and 20 μm (*K’*–*M’*). cpce, *ca8* promoter–derived PC-specific enhancer element; dpf, days post fertilization; Dyrk1A, dual-specificity tyrosine phosphorylation–regulated kinase 1A; HB, hindbrain; PCs, Purkinje cells; TeO, tectum opticum.

Recently, we have described a small PC-specific regulatory element derived from the zebrafish carbonic anhydrase 8 (*ca8*) enhancer (*ca8* promoter–derived PC-specific enhancer element [cpce]) (39, 40) that we used in tandem orientation in a bidirectional manner to coexpress (HA)-tagged human *dyrk1a* together with the membrane-targeted bright GFP Fyn-mClover (Fig. 2*A*). αHA antibody whole-mount immunostaining confirmed the coexpression of hDyrk1A and mClover in transient transgenic 4 dpf larvae (Fig. 2, *B* and *C*). Coexpression of both proteins was evident (Fig. 2*D*) such that FynmClover fluorescence can serve as both an indicator of *hdyrk1a* expression in living specimens and a reporter of morphological consequences of hDyrk1A hyperactivity on PC morphology. In addition, immunohistochemistry against parvalbumin confirmed the identity of the mClover-expressing neurons as cerebellar PCs (Fig. 2, *E*–*G*) (41).

Subsequently, we generated a stable transgenic line, designated as Tg(*ca8*-E1b:FynmClover,HA-hDyrk1A)^bz19^ or PC-Dyrk1A in short, and confirmed expression of hDyrk1A by αHA staining (Fig. 2, *H*–*J*, dorsal overview over cerebellar hemispheres) and its reliable coexpression with the membrane-tagged mClover in 5 dpf larvae from the F2 generation at higher magnification by confocal laser scanning microscopy (Fig. 2, *K*–*M*, see colocalization of mClover and anti-HA staining in insets Fig. 2, *K’*–*M’*, white arrows). These transgenic fish grow and mate properly and do not display any obvious abnormalities neither in overall brain morphology nor in behavior and cannot be distinguished from WT counterparts by gross inspection.

### Dyrk1A overexpression compromises PC-layer morphology in zebrafish larvae

**Figure 3:**
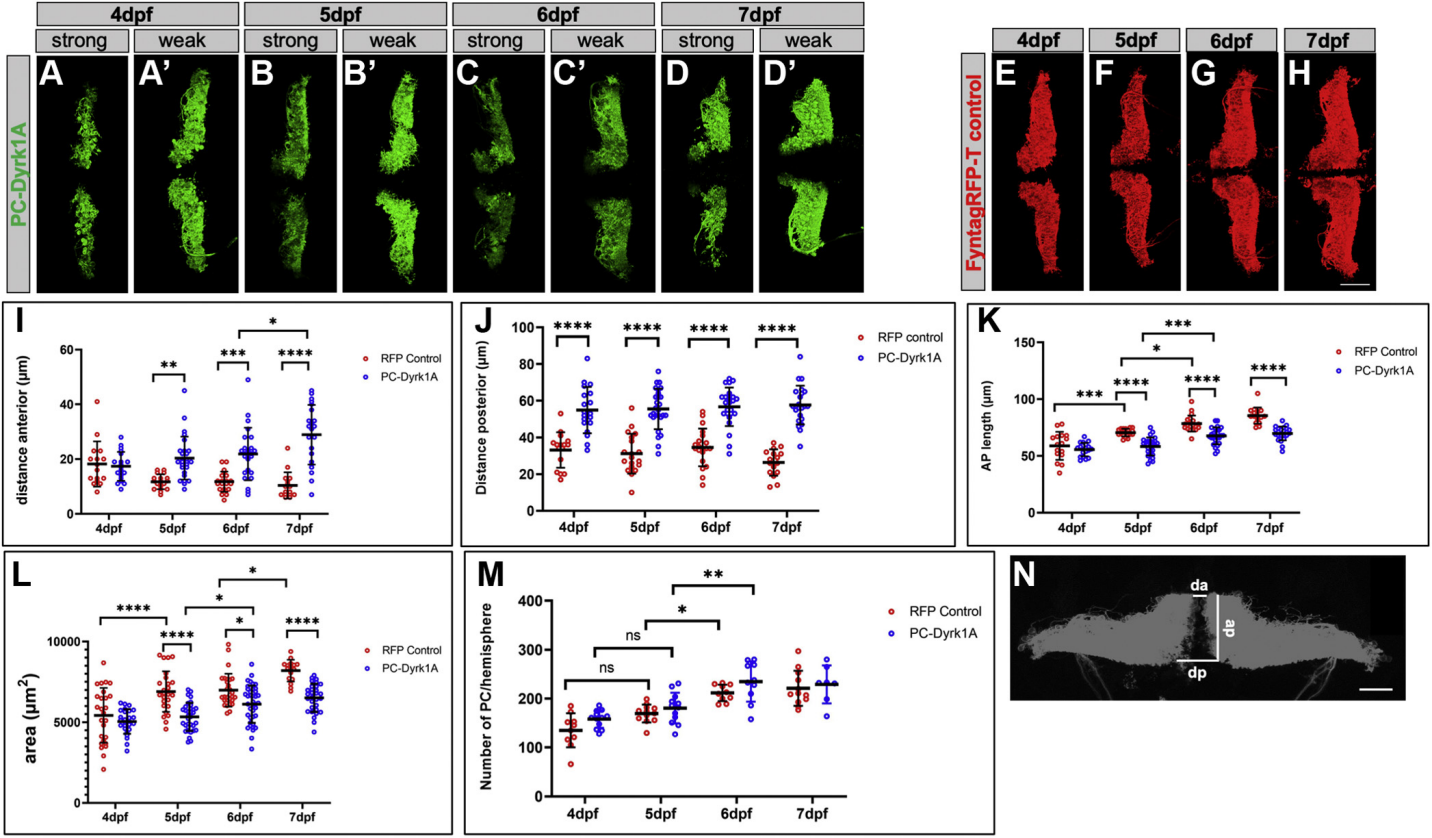
**hDyrk1A overexpression in PCs impairs morphology of the PC layer.***A*–*H*, PC layer of either PC-Dyrk1A larvae displaying *green* mClover fluorescence or PC-RFP control larvae with *red* fluorescence between 4 and 7 dpf. Dorsal view (anterior to the *left*) of left and right PC hemispheres of different PC-Dyrk1A larvae from the same developmental stage with either strong (*A*–*D*) or mild (*A’*–*D’*) phenotype in PC layer morphology and corresponding controls (*E*–*H*). *I*–*M*, quantification of morphological abnormalities from PC-RFP control (*red*) and PC-Dyrk1A (*blue*) larvae between 4 and 7 dpf (n = 12–19). Measurements of anterior (*I*) and posterior (*J*) distance between the two PC layer hemispheres, anterior–posterior length (*K*) and area (*L*) of individual hemispheres, as well as the numbers of PCs (*M*) per hemisphere. *I*–*M*, data are the mean ± SD (error bars). Statistical analysis was performed using two-way ANOVA, followed by Tukey's post hoc multiple comparisons test. *I*, interaction: F = 10.25, *p* < 0.0001; row factor: F = 1.725, *p* = 0.1642; column factor: F = 59.45. *p* < 0.0001. ∗*p* = 0.0437, ∗∗*p* = 0.0051, ∗∗∗*p* = 0.0004, and ∗∗∗∗*p* < 0.0001. *J*, interaction: F = 1.682, *p* = 0.1732; row factor: F = 0.8437, *p* = 0.4719; column factor: F = 59.45, *p* < 0.0001 and ∗∗∗∗ *p* < 0.0001. *K*, interaction: F = 4.523, *p* = 0.0045; row factor: F = 56.01, *p* < 0.0001; column factor: F = 80.41, *p* < 0.000. ∗*p* = 0.0272, ∗∗∗ RFP control 4 dpf *versus* 5 dpf RFP *p* = 0.002, ∗∗∗PC-Dyrk1A 5 dpf *versus* 6 dpf *p* = 0.0003, ∗∗∗∗ *p* < 0.0001. *L*, interaction: F = 4.035, *p* = 0.0081; row factor: F = 31.15, *p* < 0.0001; column factor: F = 56.54, *p* < 0.0001. ∗ PC-Dyrk1A 5 dpf *versus* 6 dpf *p* = 0.0486; ∗ RFP control *versus* PC-Dyrk1A 6 dpf *p* = 0.0272, ∗ RFP control 6 dpf *versus* 7 dpf *p* = 0.0104, ∗∗∗∗ *p* < 0.0001. *M*, interaction: F = 0.4441, *p* = 0.7221; row factor: F = 32.67, *p* < 0.0001; column factor: F = 6.727, *p* = 0.0113. ∗ RFP control 5 dpf *versus* 6 dpf 0.0321, ∗∗ PC-Dyrk1A 5 dpf *versus* 6 dpf *p* = 0.0017. *N*, schematic drawing to illustrate the positions of measurements quantified in panels *I*–*K*. Anterior to the *left* (*A*–*H*) and to the *top* (*N*), respectively. The scale bar represents 50 μm (*A*–*H* and *N*). ap, anterior–posterior length; da, distance anterior; dp, distance posterior; dpf, days post fertilization; Dyrk1A, dual-specificity tyrosine phosphorylation–regulated kinase 1A; PCs, Purkinje cells.

The development of the zebrafish PC layer starts at 2.5 dpf, and the layer expands in size and complexity during the following days (39, 42). Both hemispheres approach and display a characteristic wing-shaped morphology. This developmental process is recapitulated by the transgenic line Tg(*ca8*-E1B:FMATagRFP)^bz4^ (43) or PC-RFP in short (Fig. 3, *E*–*H*). The PC layer development of PC-Dyrk1A larvae, which initiates Dyrk1A overexpression upon PC differentiation, however, was severely compromised (Fig. 3, *A*–*D*). Opposite to controls, the distance between the two cerebellar hemispheres increased and the area of the PC layer appeared to be reduced between 3 and 7 dpf. Laser scanning confocal microscopy analysis of the PC layer initially revealed a milder (Fig. 3, *A*’–*D’*) and a more pronounced (Fig. 3, *A*–*D*) phenotype in larvae. Yet, the mild phenotype seemed to be an intermediate state of a progressing phenotype as the percentage of larvae with a strongly affected PC layer continuously increased from 5.3% at 4 dpf to 78.6% at 7 dpf.

To further quantify these observations, we measured the anterior distance (da) (Fig. 3*I*) and posterior distance (dp) (Fig. 3*J*) between the hemispheres, the anterior–posterior (ap) length (Fig. 3*K*), and the area covered by the PC population (Fig. 3*L*) in transgenic PC-RFP and PC-Dyrk1A larvae between 4 and 7 dpf. The average da between the PC hemispheres (reference points for measurements are shown in Fig. 3*N*) in PC-RFP control larvae decreased from 18.5 μm at 4 dpf to 12.6 μm at 5 dpf, reaching a value of 10.9 μm at 7 dpf. In contrast, in PC-Dyrk1A larvae, the average anterior hemisphere distance increased from 17.8 μm at 4 dpf to 28.4 μm at 7 dpf and was from 5 dpf onward significantly increased as compared with the respective controls (Fig. 3*I*; 5 dpf: *p* = 0.0051; 6 dpf: *p* = 0.0004; 7 dpf: *p* ≤ 0.0001). This represents a more than 2.5-fold difference between WT and PC-Dyrk1A larvae in this da at 7 dpf.

The average dp (for reference points, see Fig. 3*N*) between the hemispheres in controls remained almost constant during ongoing PC layer differentiation, with a slight, but not significant, reduction between 6 and 7 dpf (Fig. 3*J*; 34.9 μm *versus* 26.9 μm). Similarly, in PC-Dyrk1A larvae, this average dp did not vary much between subsequent days of development. But, in comparison with PC-RFP controls, this average distance was strongly increased already at 4pf and more than double in size in the PC-Dyrk1A cerebellum at 7 dpf (Fig. 3*J*; 58.1 μm *versus* 26.9 μm; *p* < 0.0001).

The extension of the PC population along the ap axis (see Fig. 3*N* for measurement reference points) gradually increased in both groups (Fig. 3*K*). PC-RFP control larvae displayed an average length of 59.3 μm at 4 dpf rising to 85.9 μm at 7 dpf with significant increases between individual days until 6 dpf. In PC-Dyrk1A larvae, the rise from an average ap length of 56.1 μm to 70.2 μm between 4 and 7 dpf was less pronounced, in comparison with PC-RFP controls this increase in length was significantly lower starting from 5 dpf onward.

Finally, the average area that was covered by the PC population was quantified. Owing to continuous growth and addition of PCs over time, the individual PC-covered hemispheres expanded in size in both PC-RFP and PC-Dyrk1A larvae (Fig. 3*L*). In PC-RFP controls, these hemispheres covered 5426 μm^2^ on average at 4 dpf, which enlarged to 8198 μm^2^ at 7 dpf. Significant increases were observed between 4 and 5 dpf (from 5426 μm^2^ to 6899 μm^2^; *p* = 0.0001) and 6 and 7 dpf (from 6986 μm^2^ to 8198 μm^2^; *p* = 0.0104).

At 4 dpf, the average area covered by a PC hemisphere in PC-Dyrk1A larvae was 5049 μm^2^ and thus nearly the same as in WT controls. With ongoing cerebellum differentiation, more space was occupied on average by the hemispheres during each successive day (5 dpf: 5335 μm^2^; 6 dpf: 6180 μm^2^, *p* = 0.0486; 7 dpf: 6602 μm^2^). Yet, this expansion is less vigorous, and by 7 dpf, the size of a hemisphere in PC-Dyrk1A is nearly 20% smaller than that in WT controls (Fig. 3*L*).

This reduced hemisphere expansion in PC-Dyrk1A larvae could reflect a reduced number of PCs. Therefore, fluorescent PCs from PC-RFP and PC-Dyrk1A were counted in individual larvae between 4 and 7 dpf on image stacks recorded by confocal microscopy. A general increase in the cell number in both the PC-Dyrk1A and the PC-RFP control larvae could be determined between 5 and 6 dpf, while the slight increase at 7 dpf was no longer significant. But interestingly, a significant difference in average PC numbers between the two groups could not be determined during the individual days of cerebellum differentiation (Fig. 3*M*). Thereby, the determined PC number and the temporal profile of PC addition to the hemispheres corresponds very well with recently published data despite the use of different counting methods (39). Consequently, cell death of PCs can be ruled out as a cause for the reduced size of hemispheres because no reduction in the number of cells in PC-Dyrk1A larvae could be detected. Instead, these findings of smaller hemispheres containing comparable cell numbers to WT larvae argue for a more compact organization of PCs in PC-Dyrk1A larvae.

In summary, overexpression of Dyrk1A in PCs does not interfere with their initial survival and differentiation, but results in a more densely organized PC layer as indicated by the smaller territory occupied by the PC layer, consisting of similar cell numbers in PC-Dyrk1A larvae than in WT counterparts.

### Dyrk1A overexpression affects swimming behavior in zebrafish larvae

**Figure 4:**
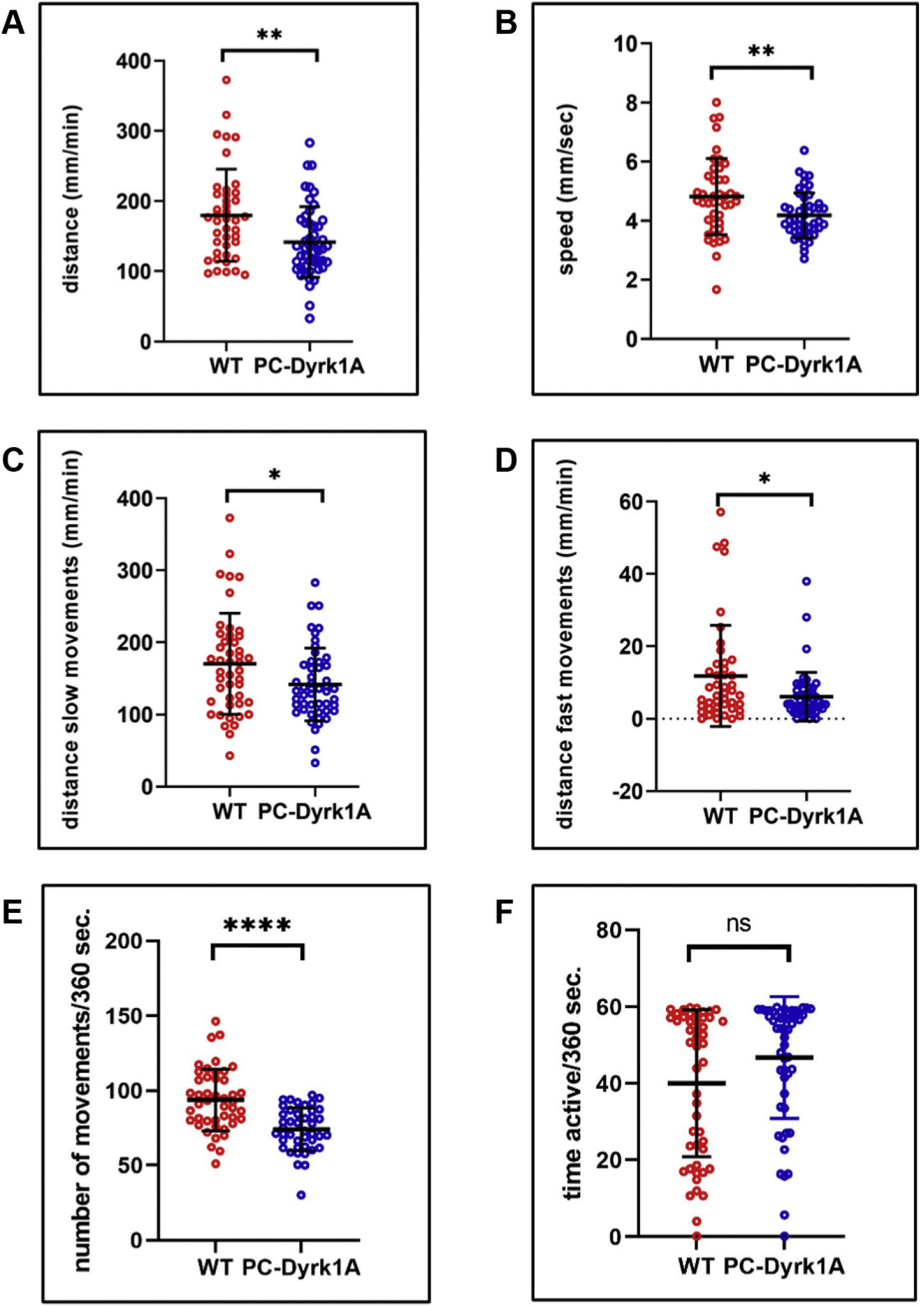
**hDyrk1A overexpression in PCs compromises swimming behavior.***A*–*D*, swimming activity of 5 dpf WT and heterozygous PC-Dyrk1A larvae; mean values ±SD of 30 min tracks. *A*, PC-Dyrk1A larvae swam significantly shorter distances than WT larvae (two-tailed unpaired *t* test, *p* = 0.022) and *B*, consequently displayed a reduced average swim speed (two-tailed unpaired *t* test, *p* = 0.0041). *C*, the distance swum with slow movements (0.2< slow speed <21 mm/s) was significantly decreased (two-tailed unpaired *t* test, *p* = 0.0228) in PC-Dyrk1A larva (*D*) as was the distance covered by fast movements (fast speed >21 mm/s, nonparametric *t* test *p* = 0.0385). The number of movements was significantly decreased in PC-Dyrk1A larva (*E*, two-tailed unpaired *t* test, *p* = 0.0012), whereas the period of activity was almost equal in both groups (*F*, nonparametric *t* test, ns, *p* = 0.0612). All values are the means ± SD (error bars). WT: n = 44; PC-Dyrk1A: n = 50; ∗*p* < 0.05; ∗∗*p* < 0.02; ∗∗∗*p* < 0.005. dpf, days post fertilization; Dyrk1A, dual-specificity tyrosine phosphorylation–regulated kinase 1A; ns, not significant; PCs, Purkinje cells.

The cerebellum is important for the control of motor skills that are necessary for coordinated and smooth movements as well as motor learning (44, 45), and PCs as principal cerebellar neurons are responsible for encoding this cerebellar output. Because PC-Dyrk1A zebrafish larvae show an altered spatial compaction of the PC layer and an increased separation between the PC hemispheres, we wondered whether this altered morphology resulted in changes in swimming behavior in open field tests in affected larvae. During a 30-min locomotion recording, WT zebrafish swum an average distance of 181.8 mm/min, while the PC-Dyrk1A group covered a significantly shorter distance of 148.2 mm/min (Fig. 4*A*). Also, the speed of swimming of PC-Dyrk1A larvae was significantly reduced (4.18 mm/s) as compared with the controls (4.82 mm/s, Fig. 4*B*). This activity was further divided in fast (>20 mm/s) and moderate (between 0.2 and 20 mm/s) swim speeds, and the distances that were covered with these movements were determined. The main distance in both groups was preferentially covered with moderate swim movements, whereby this distance was again significantly reduced for PC-Dyrk1A larvae (Fig. 4*C*). Fast swim speeds were rarely observed and only accounted for a small proportion of the total distance (6.3% for controls and 4.1% for PC-Dyrk1A, Fig. 4*D*). Again, the distance covered by fast movements was significantly reduced for PC-Dyrk1A larvae compared with controls (Fig. 4*D*).

Because zebrafish swimming at this age is discontinuous and stochastic, we also compared the number of swim events and the duration spent swimming. This analysis revealed that PC-Dyrk1A larvae with an average of 74.2 movements during 6 min displayed a lower number of movements than the controls, which performed an average of 93.8 movements during this time period (Fig. 4*E*). But, no significant difference was observed when the duration of swimming during a 6-min period was determined (40.0 s for controls and 46.8 s for PC-Dyrk1A, Fig. 4*F*). This means that PC-Dyrk1A larvae initiate swimming less often, but each swim activity lasts longer yet at a slower speed covering shorter distances.

### Disorganized PC layer in adult PC-Dyrk1A brains

**Figure 5:**
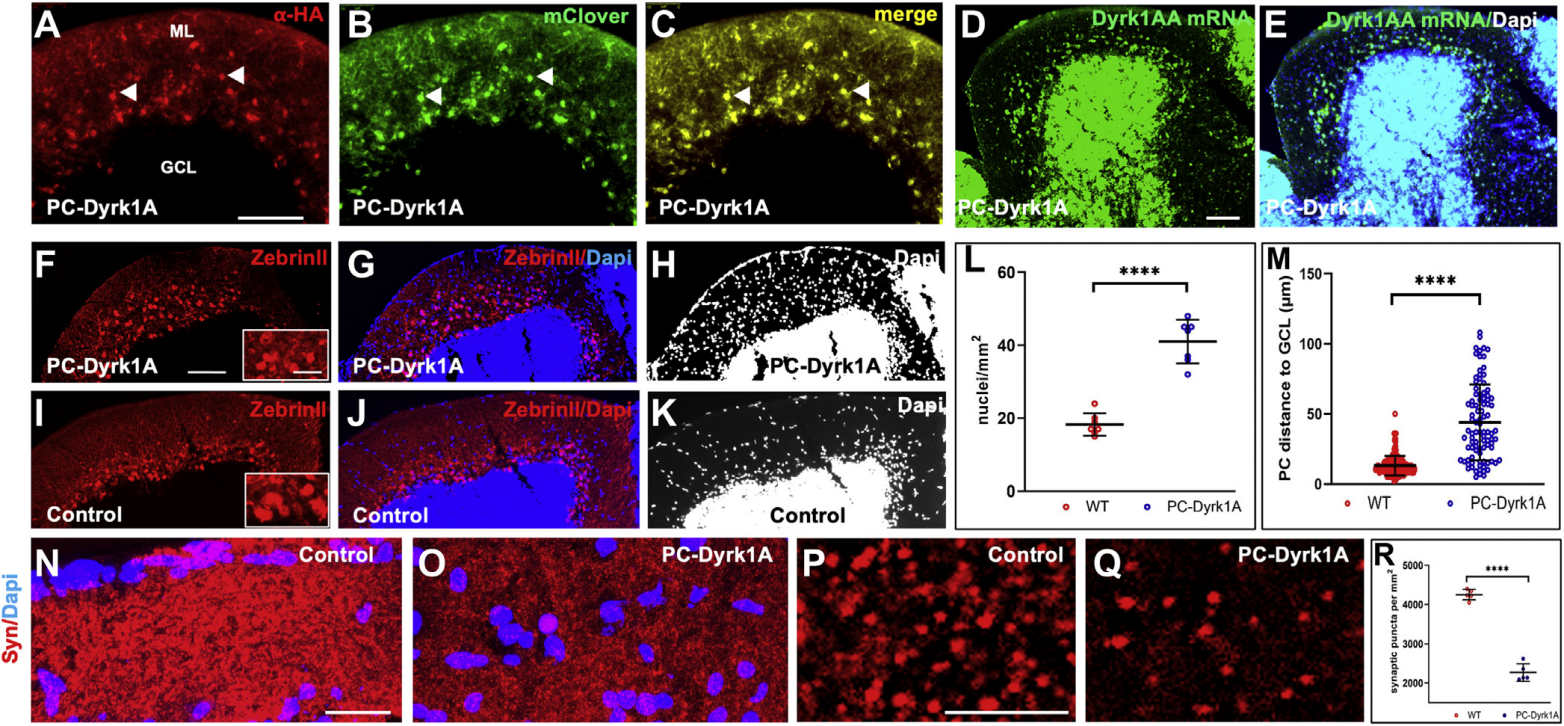
**A compromised PC layer in adult PC-Dyrk1A brains is accompanied by a strong loss of synaptic puncta.***A*–*C*, the expression of Dyrk1A and mClover persists in adult brains of PC-Dyrk1A transgenic fish as shown on frozen cerebellar sagittal sections stained with (*A*) αHA or (*B*) mClover expression. *C*, the overlay of the images documents the coexpression of hDyrk1A and the fluorescent mClover protein. *dyrk1aa in situ* hybridization (*D*) with DAPI counterstaining (*E*) demonstrated that the endogenous expression of zebrafish *dyrk1aa* is maintained in the cerebellum of adult PC-Dyrk1A fish. *F*–*K*, PC-specific ZebrinII immunohistochemistry with DAPI counterstaining on adult frozen sagittal cerebellar sections of PC-Dyrk1A (*F*–*H*) and PC-RFP controls (*I*–*K*). Purkinje cells are no longer found in an orderly fashion between granule cell layer (GCL) and the molecular layer (ML) in PC-Dyrk1A fish as in controls but appear scattered throughout the ML (*A*–*C*, *white arrowheads*). This is confirmed by quantitative analysis of (*L*) cell nucleus density in the ML and (*M*) the distance of PCs to the GCL (unpaired two-tailed *t* test, *p* < 0.0001). Compared with controls (*N* and *P*), the synaptic density in the molecular layer was reduced in PC-Dyrk1A brains (*O* and *Q*) shown by anti-Synaptophysin immunohistochemistry counterstained with DAPI and quantified by counting the density of synaptic puncta (*R*) (unpaired two-tailed *t* test, *p* < 0.0001). Values are the means ± SD (error bars). Anterior is to the *left* and dorsal up. The scale bar represents 100 μm (*A*–*K*), 20 μm (insets *F* and *I*), 20 μm (*N* and *O*), and 5 μm (*P* and *Q*). DAPI, 4’,6-diamidino-2-phenylindole; GCL, granule cell layer; ML, molecular layer. ∗∗∗∗*p* < 0.0001.

Because the cpce continues to express transgenes until adulthood (39), we wondered about the long-term effects of Dyrk1A overexpression in cerebellar PCs. We therefore performed immunohistochemistry on sagittal sections of the adult cerebellum of heterozygous PC-RFP and PC-Dyrk1A zebrafish. αHA-stained hDyrk1a and mClover expression indeed indicated the maintenance of transgene expression and displayed a clear colocalization (Fig. 5, *A*–*C*). mRNA *in situ* hybridization against zebrafish *dyrk1aa* confirmed that hDyrk1A expression did not affect continuous endogenous *dyrk1aa* expression (Fig. 5, *D* and *E*).

Surprisingly, however, PCs in PC-Dyrk1A zebrafish were randomly distributed throughout the molecular layer (ML) instead of localizing to the PC layer embedded between the GCL and ML. This abnormal distribution was confirmed by ZebrinII immunohistochemistry as an independent PC antigen (Fig. 5, *F* and *I*).

Consequently, we observed a much higher number of 4’,6-diamidino-2-phenylindole–positive nuclei in the ML as compared with control sections (Fig. 5, *G*, *H*, *J* and *K*). Analyses revealed a 2.3-fold increase in nuclei positioned in the ML in PC-Dyrk1A brains (Fig. 5*L*; WT: 18.3 *versus* PC-Dyrk1A: 41.0, *p* < 0.0001) likely because of the misplaced PCs that were on average about 3.3-fold further apart from the GCL compared with the controls (Fig. 5*M*; WT: 13.2 μm *versus* PC-Dyrk1A: 44.0 μm, *p* < 0.0001).

To address whether the misplacement of PCs in PC-Dyrk1A adult brains would manifest synaptic ramifications, we performed immunohistochemistry with the presynaptic marker synaptophysin (Syn). Compared with PC-RFP controls (Fig. 5*N*), the synaptic density in the ML of PC-Dyrk1A brains (Fig. 5*O*) was strongly diminished. The quantification of Syn puncta derived from image recording at higher magnification from control brains (Fig. 5*P*) compared with PC-Dyrk1A brains (Fig. 5*Q*) showed a strong and significant reduction of synaptic puncta diminishing to about half of the WT density (Fig. 5*R*, WT: 4254 ± 135, 5.3/μm^2^ *versus* PC-Dyrk1A: 2268 ± 225, 2.8/μm^2^, n = 5).

### Aged adult PC-Dyrk1A zebrafish show a compromised swimming behavior

The PC phenotype in adult PC-Dyrk1A brains prompted us to investigate the swimming behavior. While adult PC-Dyrk1A fish appeared to swim grossly indistinguishably from WT, aged PC-Dyrk1A zebrafish older than 16 months displayed occasionally rotations around their body axis and looping upon acute stress, but otherwise they seemed to stay close to the bottom of their tank. Interestingly, adult zebrafish homozygous for a nonfunctional mutant *dyrk1aa* allele have been found to exert increased exploratory, anxiolytic behavior in the novel tank test (24). In this test, zebrafish, when transferred to a new tank, initially remain at the bottom of this new environment and only after some delay start to gradually explore higher areas in the water column, which can be quantified by the number of zone transitions into the upper half of the novel tank and the time spent in this elevated area (46). Long dwell times in the bottom zone are interpreted as anxiety-like behavior but could also reflect compromised locomotor function.

**Figure 6:**
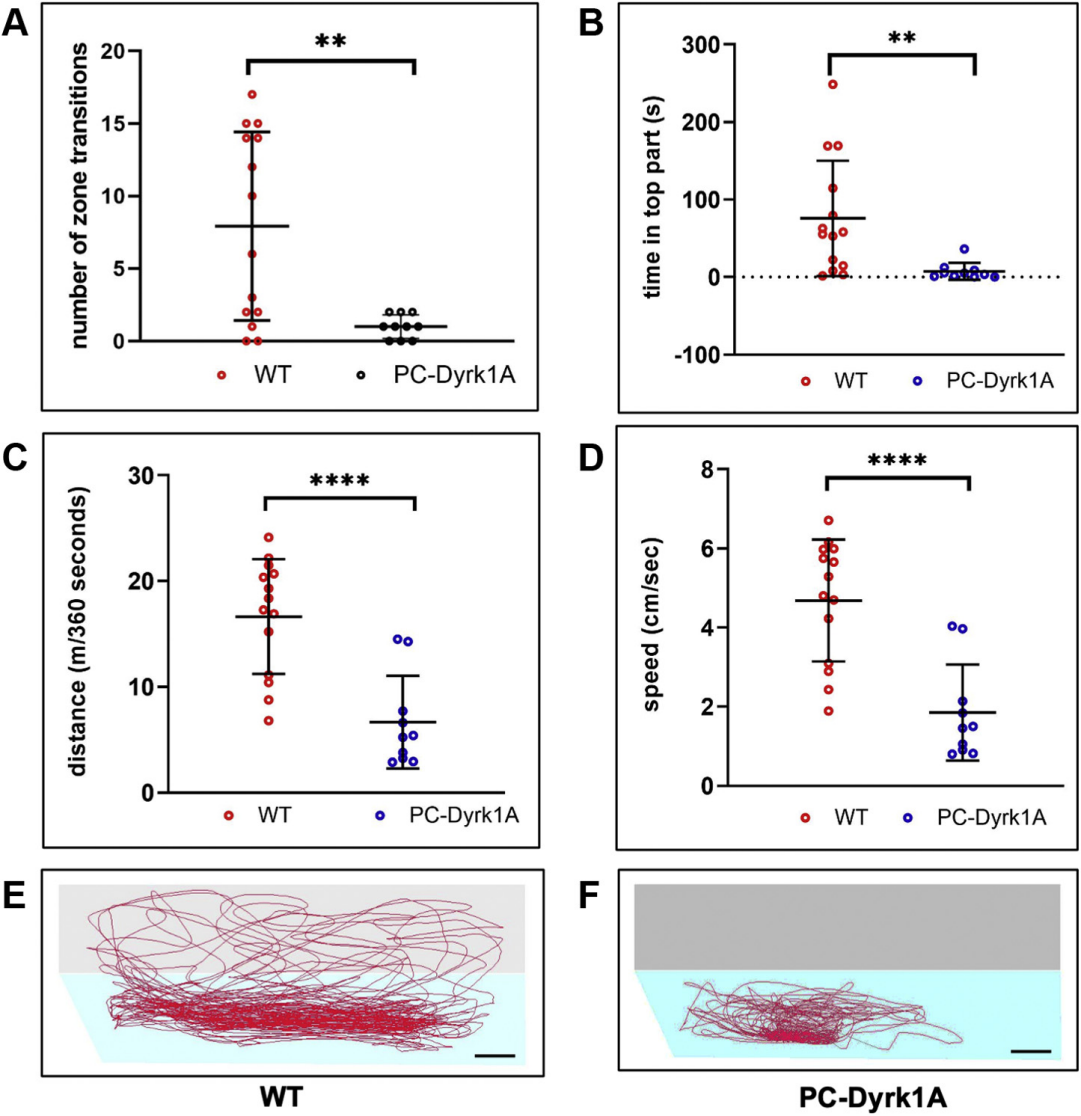
**Novel tank diving test reveals anxiety-like behavior in adult *Tg(Car8-hDryk1A-mClover)* fish.***A*–*F*, swimming behavior of adult WT (n = 14) and PC-Dyrk1A fish (n = 10) examined in the novel tank diving test. The number of zone transitions (*A*, two-tailed unpaired *t* test, *p* = 0.003), the time in the top part (*B*, nonparametric *t* test, *p* = 0.0015), the distance covered (*C*, two-tailed unpaired *t* test, *p* < 0.0001), and the speed (*D*, two-tailed unpaired *t* test, *p* < 0.0001) were determined in 6-min recordings. All parameters analyzed were significantly reduced in PC-Dyrk1A fish. *E* and *F*, representative tracks of a control (*E*) and PC-Dyrk1A zebrafish with bottom (*light blue*) and *top part* (*light gray*) of the tank. Means ± SD. The scale bar represents 3 cm. Dyrk1A, dual-specificity tyrosine phosphorylation–regulated kinase 1A; PCs, Purkinje cells. ∗∗*p* < 0.01, ∗∗∗∗*p* < 0.0001.

Aged PC-Dyrk1A zebrafish older than 16 months displayed clear abnormalities in their swimming behavior in this novel tank test compared with age-matched WT controls. Both the number of transitions into the upper zone (Fig. 6*A*) and the dwell time in the upper part were significantly reduced (Fig. 6*B*). Like the PC-Dyrk1A larvae, the aged adult fish also covered a significantly shorter average swim distance (Fig. 6*C*) and swam at a lower average speed than the controls (Fig. 6*D*). Representative tracks of WT (Fig. 6*E*) and PC-Dyrk1A (Fig. 6*F*) fish further demonstrated that the swimming movements of the PC-Dyrk1A fish were carried out within a limited area.

Together with the neuroanatomical defects in cerebellar neuroanatomy, these findings support the concept that developmental defects caused by Dyrk1A hyperactivity last until adulthood and likely progress into the disintegration of the well-ordered PC layer, accompanied by a reduction in synaptic contacts of PCs, which negatively impacts on swimming activity. Whether this behavior is caused by deficits in locomotor control, increased anxiety, or both needs to be further investigated.

### Dyrk1A inhibitors antagonize the structural disorganization of the PC layer in PC-Dyrk1A zebrafish larvae

**Figure 7:**
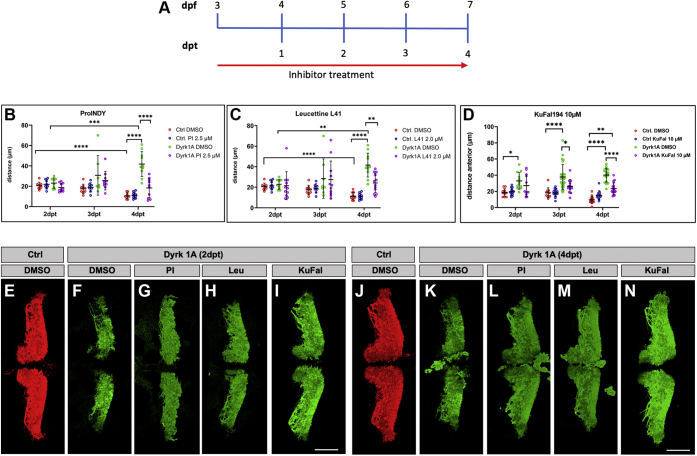
**Dryk1A inhibitors rescue the morphological Purkinje cell layer impairments of PC-Dyrk1A larvae.***A*, scheme of inhibitor administration. Inhibitors dissolved in DMSO were diluted to the respective concentrations in 30% Danieau. 3 dpf larvae were treated for 4 days, and the inhibitor was changed daily. *In vivo* imaging of PC hemispheres of all groups was performed between 2 and 4 dpt. *B*–*D*, measurements of the anterior distance (μm) of the PC hemispheres for (*B*) ProINDY, (*C*) Leucettine L41, and (*D*) KuFal194 treated larvae. Each inhibitor test encompasses the following groups: PC-RFP control larvae treated with DMSO (Ctrl, DMSO), PC-RFP control larvae treated with inhibitor (Ctrl + inhibitor), PC-Dyrk1A larvae treated with DMSO (Dyrk1A DMSO), and PC-Dyrk1A larvae treated with inhibitor (Dyrk1A + inhibitor). *B*–*D*, data are the mean ± SD (*error bars*). Statistical analysis was performed using three-way ANOVA, followed by Tukey's post hoc multiple comparisons test. *B*, row factor: F = 0.9372 *p* = 0.3953, row factor (Ctrl DMSO Ctrl PI *versus* Dyrk1A DMSO Dyrk1A PI): F = 0.9372, *p* = 0.3953; row factor (Ctrl DMSO Dyrk1A DMSO *versus* Ctrl PI Dyrk1A PI): F = 10.47, *p* = 0.0017; row factor x (Ctrl DMSO Ctrl PI *versus* Dyrk1A DMSO Dyrk1A PI): F = 12.75, *p* < 0.0001; row factor x (Ctrl DMSO Dyrk1A DMSO *versus* Ctrl PI Dyrk1A PI): F = 4.843, *p* = 0.0099; row factor (Ctrl DMSO Ctrl PI *versus* Dyrk1A DMSO Dyrk1A PI) x (Ctrl DMSO Dyrk1A DMSO *versus* Ctrl PI Dyrk1A PI): F = 14.47, *p* = 0.0002; row factor x (Ctrl DMSO Ctrl PI *versus* Dyrk1A DMSO Dyrk1A PI) x (Ctrl DMSO Dyrk1A DMSO *versus* Ctrl PI Dyrk1A PI): F = 3.555, *p* = 0.0324. ∗∗∗*p* = 0.0003 and ∗∗∗∗*p* < 0.0001. *C*, row factor: F = 0.1570, *p* = 0.8549; row factor (Ctrl DMSO Ctrl L41 *versus* Dyrk1A DMSO Dyrk1A L41): F = 30.85, *p* < 0.0001; row factor (Ctrl DMSO Dyrk1A DMSO *versus* Ctrl L41 Dyrk1A L41): F = 2.139, *p* = 0.1466; row factor x (Ctrl DMSO Ctrl L41 *versus* Dyrk1A DMSO Dyrk1A L41): F = 9.839., *p* = 0.0001; row factor x (Ctrl DMSO Dyrk1A DMSO *versus* Ctrl L41 Dyrk1A L41): F = 2.441, *p* = 0.0921; row factor (Ctrl DMSO Ctrl L41 *versus* Dyrk1A DMSO Dyrk1A L41) x (Ctrl DMSO Dyrk1A DMSO *versus* Ctrl L41 Dyrk1A L41): F = 3.689, *p* = 0.0575; row factor x (Ctrl DMSO Ctrl L41 *versus* Dyrk1A DMSO Dyrk1A L41) x (Ctrl DMSO Dyrk1A DMSO *versus* Ctrl L41 Dyrk1A L41): F = 1.708, *p* = 0.1863. ∗∗Dyrk1A DMSO 2 dpt *versus* 4 dpt *p* = 0.0068; ∗∗Dyrk1A DMSO *versus* Dyrk1A L41 4 dpt *p* = 0.0065; ∗∗∗∗ *p* < 0.0001. *D*, row factor: F = 1.843, *p* = 0.1617; row factor (Ctrl DMSO Ctrl KuFal194 *versus* Dyrk1A DMSO Dyrk1A KuFal194): F = 108.3, *p* < 0.0001; row factor (Ctrl DMSO Dyrk1A DMSO *versus* Ctrl KuFal194 Dyrk1A KuFal194): F = 10.35, *p* = 0.0016; row factor x (Ctrl DMSO Ctrl KuFal194 *versus* Dyrk1A DMSO Dyrk1A KuFal194) F = 3.088, *p* = 0.0482; row factor x (Ctrl DMSO Dyrk1A DMSO *versus* Ctrl KuFal194 Dyrk1A KuFal194): F = 0.5474, *p* = 0.5795; row factor (Ctrl DMSO Ctrl KuFal194 *versus* Dyrk1A DMSO Dyrk1A KuFal194) x (Ctrl DMSO Dyrk1A DMSO *versus* Ctrl KuFal194 Dyrk1A KuFal194): F = 23.42, *p* < 0.0001; row factor x (Ctrl DMSO Ctrl KuFal194 *versus* Dyrk1A DMSO Dyrk1A KuFal194) x (Ctrl DMSO Dyrk1A DMSO *versus* Ctrl KuFal194 Dyrk1A KuFal194): F = 2.762, *p* = 0.0661. ∗Ctrl DMSO *versus* Dyrk1A DMSO 2 dpt *p* = 0.0120; ∗Dyrk1A DMSO *versus* Dyrk1A KuFal 10 μM 3 dpt *p* = 0.0111; ∗∗*p* = 0.0013; ∗∗∗∗*p* < 0.0001. *E*–*N*, representative images of PC hemispheres of reconstructed confocal laser scanning z-stacks for (*E* and *J*) PC-RFP DMSO controls, (*F* and *K*) PC-Dyrk1A DMSO-treated larvae, (*G* and *L*) ProINDY-treated larvae, (*H* and *M*) Leucettine L41-treated larvae, and (*I* and *N*) KuFal194-treated larvae. *E*–*I*, 2 dpt and (*J*–*N*) 4 dpt. The scale bar represents 50 μm (*E*–*N*). dpt, days post treatment; DMSO, dimethyl sulfoxide; Dyrk1A, dual-specificity tyrosine phosphorylation–regulated kinase 1A; PCs, Purkinje cells.

Given the involvement of Dyrk1A in severe and common neurological diseases, a number of efforts have been put into developing competitive small chemical inhibitors against Dyrk kinase activity, and several of such inhibitor compounds are currently available. We therefore aimed at investigating whether such Dyrk inhibitors can reverse the structural disorganization of the PC layer in PC-Dyrk1A larvae. We decided to use the second-generation (the more recently developed) inhibitors ProINDY and Leucettine L41 because of their low cytotoxicity and bioavailability. Computational docking of these inhibitors with homology models of human Dyrk1A and zebrafish Dyrk1Aa and Dyrk1Ab suggested similar binding of the ATP-competitive inhibitors to these ATP-binding sites compared with human Dyrk1A (Figs. S2 and S3). Therefore, previously established concentrations for *in vivo* treatments of Dyrk1A hyperactivity in *Drosophila* and mouse were used (47, 48). PC-RFP and PC-Dyrk1A zebrafish larvae were treated for four consecutive days either with ProINDY (2.5 μM) or Leucettine L41 (2 μM) starting at 3 dpf when PCs begin to differentiate and express hDyrk1A (Fig. 7*A*). On each day of treatment, the PC hemispheres were documented by confocal microscopy to quantify the da between hemispheres, which displayed the strongest phenotypic deviation in PC-Dyrk1A larvae compared with controls (Fig. 3*I*).

PC-RFP larvae treated with either dimethyl sulfoxide (DMSO) or inhibitor displayed the characteristic proximation of the two cerebellar PC hemispheres from 2 to 4 days post treatment (dpt), and inhibitor-treated larvae did not show statistically significant differences to DMSO controls (Fig. 7, *B* and *C*) on 4 dpt, indicating that the applied inhibitor concentrations did not cause an obvious adverse effect on cerebellar development. Strikingly, both inhibitors were able to rescue the Dyrk1A overexpression phenotype in PC-Dyrk1A larvae by counteracting the enlarged distance of the PC hemispheres observed in DMSO-treated PC-Dyrk1A fish and by promoting PC hemisphere narrowing toward the dorsal midline at 4 dpt (Fig. 7, *B*, *C*, *E*–*H*, *J*–*M*). These results show that both tested Dyrk1A inhibitors are able to access the cerebellum and to revert neuronal phenotypes despite the challenge of a secluded blood–brain barrier. In addition, both compounds do not pose obvious toxic side effects, are tolerated well, and can be dosed properly to avoid the conversion of a Dyrk1A hyperactivity phenotype into a hypoactivity one.

The Dyrk kinase family is structurally highly related, and therefore, ProINDY as well as Leucettine L41 are not Dyrk1A-specific, but also interfere with Dyrk1B, Dyrk2, and CLK functions (20, 21). The most-selective Dyrk1A inhibitor known so far, KuFal194, has only been tested in cultured cells (22), where it showed a prominent inhibition of Dyk1A activity. Its poor solubility and mediocre cellular uptake, however, questions whether this compound would be suitable for *in vivo* applications. We therefore compared KuFal194 to the activity of ProINDY and Leucettine L41 in our PC-Dyrk1A model. PC-RFP zebrafish larvae treated with 10 μM KuFal194 showed the typical proximation of PC hemispheres at 4 dpt (Fig. 7*D*) similar as observed with ProINDY and Leucettine L41 at 4 dpt (Fig. 7, *B* and *C*). This suggests that KuFal194 is tolerated well at the used concentration and does not pose obvious adverse effects, and neither gross malformations nor differences in viability of treated larvae were observed.

In PC-Dyrk1A larvae treated with KuFal194, a clear rescue of PC hemisphere disorganization could be observed compared with DMSO controls (average PC hemisphere distance at 4 dpt: DMSO: 40.2 μm, KuFal194: 23.4 μm) that rescued the cerebellar phenotype to a similar extent as obtained with ProINDY and Leucettine L41 (average PC hemisphere distance at 4 dpt: ProINDY: 18.5 μm, Leucettine L41: 24.2 μm). Interestingly, with 10 μM KuFal194, a significant rescue was already obtained 1 day earlier, already after 3 days of inhibitor treatment, compared with ProINDY and Leucettine L41. We therefore reduced the concentration of KuFal194 to 5 μM and obtained a similar yet slower rescue, in which a significant rescue in cerebellar hemisphere distance was obtained at 4 dpt (Fig. S4). This shows that the rescue activity of KuFal194 is concentration dependent.

Confocal microscopy analysis of the cerebellar PC layer of inhibitor-treated PC-Dyrk1A larvae at 2 dpt and 4 dpt further illustrated the progredient time-dependent rescue of PC hemisphere organization (Fig. 7, *E*–*N*). Compared with PC-Dyrk1A DMSO controls (Fig. 7, *F* and *K*), the shape and extent of both PC hemispheres in ProINDY-, Leucettine L41-, and KuFal194-treated larvae was improved 2 dpt (Fig. 7, *G*–*I*) and almost completely rescued in 4 dpt larvae (Fig. 7, *J*–*N*). This demonstrates that KuFal194 has a rescue activity comparable with known Dyrk kinase inhibitors and is able to revert phenotypes in the central nervous system *in vivo*. Its superior specificity, however, makes it a more precise interventional drug against Dyrk1A hyperactivity and motivates studies on further improving its cellular uptake properties.

## Discussion

Dyrk1A is a protein kinase able to autophosphorylate tyrosine residues as well as serine/threonine residues in substrates. Belonging to the CMGC family of protein kinases, Dyrk1A is closely related to other members of the Dyrk family as well as to the CLKs. Dyrk1A has received particular attention because of its involvement not only in DS but also in several types of cancer, in which this kinase has been implicated in proliferation enhancement and reduction of cell death providing resistance to proapoptotic stimuli by chemotherapy or radiotherapy (49, 50). Hence, the characterization of Dyrk1A function has received great attention, and this kinase has been implicated in a number of cell biological processes.

Dyrk1A is expressed throughout all stages of embryonic development (25) and mutant analysis revealed major consequences on nervous system organization. This function of Dyrk1A in brain development has been investigated in different animal models, and it was shown that both hypoexpression and hyperexpression resulted in neurological phenotypes. Haploinsufficiency or heterozygous loss of function mutations in mouse and *Drosophila* mutants cause microcephaly, developmental delays, motor deficits, and autism (1, 51, 52, 53). Widespread overexpression of *dyrk1A* in mouse models led to compromised neuronal morphology in the brain as well as to cognitive and behavioral impairments recapitulating the phenotypes seen in DS patients (3, 35, 36, 54). These similar phenotypes are based on neurogenesis control by Dyrk1A. While overexpression of Dyrk1A results in premature cell cycle exit of neuronal progenitors, reduced expression of Dyrk1A generates a surplus of immature neuronal progenitors, which later fail to survive because of impairment in proper differentiation.

Beyond neurogenesis, Dyrk1A clearly exerts a number of functions in differentiating and mature neurons. We have therefore studied the expression of the *dyrk1a* homologs in zebrafish at later stages of central nervous system maturation. Here, besides expression in neurogenic zones, *dyrk1aa* and *dyrk1ab* were found to be expressed prominently in cerebellar tissues and its associated structures such as the TL. This may explain the association of altered Dyrk1A activity with cerebellum-associated diseases such as autism spectrum disorder (24) and locomotive difficulties, motor learning impairment, and reduced exploratory behavior in children diagnosed with DS (55, 56). To address consequences of *dyrk1a* hyperactivity, we have targeted a single cell type of the cerebellum, the PCs, as the principal cerebellar output neuron with human Dyrk1A overexpression. This resulted in obvious morphological changes of the structural organization of both cerebellar hemispheres and their PC populations. This phenotype is unlikely to be caused by neurogenesis defects of cerebellar PC precursors, as overexpression of Dyrk1A in our model is activated by the *ca8* regulatory element, which drives expression only in postmitotic PCs (39), and PC numbers in PC-Dyrk1A zebrafish larvae did not deviate from WT controls. Rather, a compaction of PCs within the PC layer was found, probably because of an immature dendritic arborization and thus reduced space requirements of these cells (not shown) and a reduced synaptic density. Similar observations have been made in the cerebellum in mouse DS models with a modified expression dose of *dyrk1A* (57) and in humans suffering from AD (58). Our findings propose that these neuroanatomical deficits of the cerebellar PC hemispheres with a reduced synaptic density likely occur in a cell autonomous manner and is not caused by non–cell autonomous effects, such as altered neuronal network activity, as Dyrk1A overexpression is selectively targeted to PCs in our zebrafish model.

Furthermore, an age-dependent progredient structural disorganization of cerebellar morphology was observed by PCs, leaving the orderly organization of their PC layer and becoming translocated into the above ML. Signs of inflammation or neurodegeneration could not be observed in the cerebellum of PC-Dyrk1A zebrafish (not shown). These intriguing cellular changes were accompanied by behavioral worsening of slightly reduced locomotive activity already observed in larvae, which was pronounced in aged fish older than 16 months and accompanied by a lack of exploration activity in the water column. Although this reduced exploratory activity in a novel environment could be a secondary consequence of deficits in motor coordination, it is intriguing that this phenotype is complementary to adult zebrafish homozygous for a loss-of-function allele of *dyrk1aa*. These zebrafish with lack of Dyrk1A function in all its expression domains display an increased exploratory behavior, which has been interpreted as a sign of reduced anxiety (24). It will be interesting to study whether this increase in exploratory behavior could be recapitulated by a PC-specific loss of Dyrk1A function and if PC-Drk1A fish crossed to this mutant are able to reduce or revert the hyperactive swimming observed in these mutants.

Recently, reports accumulated that neurological defects observed in mouse and *Xenopus* Dyrk1A overexpression models can be rescued, if the kinase activity is reduced by Dyrk inhibitors (14, 20, 47, 59, 60). These findings could be expanded to our PC-specific Dyrk1A overexpression model, in which cerebellar hemisphere disorganization was rescued upon treatment with two known Dyrk1A inhibitors ProINDY and Leucettine L41. These findings show that despite the sensitivity of animals to the gene copy number and thus dosage of Dyrk1A activity, Dyrk1A inhibition can be adjusted well *in vivo* and is effective in correcting central nervous system phenotypes at developmental stages when a blood–brain barrier is present (61, 62). Moreover, these inhibitors are tolerated well as we could not observe any obvious effect on morphology, survival, or behavior of treated zebrafish larvae. This makes Dyrk1A a promising druggable target for pharmaceutical research.

Yet, although these compounds belong to the most advanced Dyrk1A inhibitors and act as ATP competitors, they are not selective for Dyrk1A but interfere also with related Dyrk and CLK kinases (20, 21, 47, 63). Given the involvement of these Dyrk and CLK kinases in cell proliferation, cell cycle control, and tumor development, inhibitors need to be as selective as possible for their respective target. KuFal194 was recently developed and reported to be the most-selective Dyrk1A inhibitor to date. However, its poor solubility in aqueous solutions and mediocre cellular uptake (22, 23) questioned its efficacy in specifically lowering Dyrk1A hyperactivity *in vivo*. In our hands, KuFal194 was tolerated well by zebrafish larvae in bath applications. Strikingly, when we tested KuFal194 for its potential to revert the PC structural disorganization in PC-Dyrk1A zebrafish larvae, it proved as effective as ProINDY and Leucettine L41 albeit at 2- to 4-fold higher concentration of 5 and 10 μM. Yet, this lower uptake from water might be overcome if KuFal194 is administered as a food additive. These findings show that the selective Dyrk1A inhibitor KuFal194 is a promising starting point for the development of therapeutics to mitigate neurological deficits in such severe diseases as DS. Furthermore, our PC-Dyrk1A zebrafish model represents a useful model for testing and validating Dyrk1A inhibitors by quantifiable means providing simultaneously insights into potential side effects and toxicity in a living vertebrate.

## Experimental procedures

### Animal husbandry

Zebrafish were maintained and raised at 28 °C on a 4:10 h light:dark cycle according to standard protocols (64, 65). Experiments were carried out with specimens from the AB WT or *brass* zebrafish strains. Natural mating was used to obtain embryos and larvae, and staging was performed according to dpf (66). Zebrafish embryos were incubated in egg water (0.03% g/l sea salt) for 6 h and then kept in Danieau medium (0.12 mM MgSO_4_, 0.21 mM KCl, 0.18 mM Ca(NO_3_)_2_, 17.4 mM NaCl, 1.5 mM Hepes, pH 7.2). For *in situ* hybridizations, immunohistochemistry and live imaging experiments, Danieau was supplemented with 0.005% phenylthiourea (Sigma-Aldrich) to suppress pigmentation. The following lines were used in this study: Tg(*ca8*-E1B:FMATagRFP)^bz4^ (43), and Tg(*ca8*-E1b:FynmClover,HA-hDyrk1A)^bz19^.

### Isolation of zebrafish *dyrk1aa* and *dyrk1ab* cDNAs

RNAPure peqGOLD (PEQLAB Biotechnologie GmbH) was used to isolate total RNA from 4 dpf embryos. Subsequently, oligo(dT) (Promega) primed cDNA was synthesized with avian myeloblastosis virus reverse transcriptase from 2 μg total RNA. Zebrafish *dyrk1aa*- and *dyrk1ab*-specific *in situ* probes were PCR-amplified with the following primers:

*dyrk1aa* (551 bp): sense: CAAGACAGGCGTTTGTGC; antisense: GAATCGGCCTCCTCATCTAGATCAGGAGCTGG

*dyrk1ab* (671 bp): sense AGCAGCGGGCATTTCGGCACA; antisense: TTCAGCTAAAGTCACGAGCTGGCCATCGAG

Dyrk1aa and Dyrk1ab cDNA for the generation of *in situ* probes were amplified from the 3’ region of the genes. The identity of both probes is about 40% with numerous gaps within homologous nucleotide sequences. Owing to highly stringent hybridization conditions at 68 °C, cross reactivity can be excluded.

### Cloning

For synthesis of digoxigenin (DIG)-UTP– or fluorescein-UTP–labeled *dyrk1aa* and *dyrk1ab* sense or antisense RNA probes, the respective PCR cDNA fragments were cloned into the pGEM-T Easy vector (Promega) and transcribed with T7 or SP6 RNA polymerase.

A pB-Tol2 vector consisting of two cpce (258 bp) dimers described in (39) were used in opposite orientation flanked by E1B promoters on each side. On the left side, a GFP Fyn-mClover starting with a membrane targeting sequence (MGCVQCKDKEATKLT-ST-mClover) derived from Fyn kinase was inserted into the *EcoR*V/*Kpn*I restriction sites. In the multiple cloning site of the right construct arm, a HA-hDyrk1A *Cla*1-blunt *Not*I fragment was introduced into the *EcoR*I-blunt *Not*I sites of the vector (Fig. 1*A*). The resulting transgene Tol2_pA Fyn mClover_E1B_4xcpce_E1B_Ha-hDyrk1A pA_Tol2 is designated as Tg(*ca8*-E1b:FynmClover,HA-hDyrk1A)^bz19^ or PC-Dyrk1A. The HA-hDyrk1A containing the human *dyrk1a* cDNA was a kind gift from W. Becker.

### Generation of transient and stable transgenic zebrafish

Transgenic embryos were generated by coinjecting plasmid DNA (25 ng/μl) and TOL2 transposase mRNA (25 ng/μl) into one-cell stage embryos (67, 68). Fluorescent transient transgenic larvae were analyzed between day 3 and day 7 of development. Stable *ca8*-E1b:FynmClover,HA-hDyrk1A transgenics were generated from embryos with broad PC-specific fluorescence, which were grown to adulthood and screened for stable integration in their offspring. Initially, 15 F0 founders were identified; three expressing founders with strong and faithful expression of mClover and hDyrk1A were used to establish independent F1 families. As these showed comparable phenotypes, one transgenic line was eventually continued, which is now in the F3 generation.

### Whole-mount *in situ* hybridization

Whole-mount *in situ* hybridization on zebrafish embryos and larvae was performed as described previously with slight modifications (69). Briefly, embryos and larvae were fixed in 4% paraformaldehyde (PFA)/phospahte buffered saline (PBS) overnight, dehydrated in 25%, 50%, and 75% methanol/PBS and stored in 100% methanol at −20 °C overnight or until use. The embryos were rehydrated, permeabilized in 10 μg/ml proteinase K for 5 to 120 min depending on their developmental stage, and postfixed for 20 min in 4% PFA followed by three PBS-Tween20 (PBS-T) washing steps. Prehybridization was performed in 50% formamide, 5x saline sodium citrate (SSC), 9.2 mM citric acid, pH 6.0, 50 μg/ml heparin, 50 μg/ml yeast tRNA, and 0.1% Tween-20 for at least 1 h at 65 °C followed by hybridization with antisense probe overnight. Next, the embryos were washed 2× 45 min in 50% formamide/2× SSC/0.1% Tween-20, 1× 45 min in 2× SSC/0.1% Tween-20, and 2× 45 min in 0.2× SSC/0.1% Tween-20 at 68 °C. For probe detection, the specimens were blocked in 1× blocking solution (1% blocking reagent, Sigma-Aldrich) in maleic acid buffer (0.1 M maleic acid, 0.15 M NaCl) for at least 1 h and then incubated with anti-DIG-AP antibody (1:2000 in 1× blocking solution) overnight at 4 °C. The next day, embryos were washed six times with PBS-T for 20 min and three times with alkaline phosphatase buffer (NTMT) (100 mM NaCl; 100 mM Tris, pH 9.5; 50 mM MgCl_2_; 0.1% Tween-20) for 10 min followed by staining with BM-Purple (Sigma-Aldrich).

For tissue sections, stained embryos and larvae were postfixed in 4% PFA, washed with PBS several times, and subsequently incubated overnight in 30% sucrose/PBS at 4 °C for cryoprotection. The embryos were then embedded in O.C.T. media (Tissue-Tek, Sakura Finetek) and processed for transversal 18-μm cryosections using a Leica CM3050 S cryostat (Leica Biosystems GmbH).

### *In situ* hybridization on frozen sections

Adult zebrafish were euthanized with Tricaine MS-222 (Sigma-Aldrich). Brains were dissected and fixed in 4% paraformaldehyde/PBS at 4 °C overnight. The tissue was then washed several times in PBS and equilibrated overnight in 30% sucrose/PBS at 4 °C. After a 20-min incubation in 50% sucrose/O.C.T. and two washing steps in 100% O.C.T., brains were embedded in O.C.T. using cryomolds and stored at −80 °C. Cryosections (18 μm) generated with Leica cryostat (Leica Biosystems GmbH) were rehydrated in PBS and subsequently postfixed in 4% PFA/PBS for 10 min. After quenching endogenous peroxidases for 10 min with 3% H_2_O_2_/PBS followed by three PBS washing steps, the sections were acetylated (0.1 M tri-ethanolamine/0.25% acetic anhydride) for 10 min. The sections were again washed three times with PBS and permeabilized with 0.5% Triton X-100/PBS. After prehybridization for 2 h, the solution was replaced by the hybridization solution (50% formamide, 5x SSC, 5% dextran sulfate, 50 μg/ml heparin, 0.5 mg/ml yeast tRNA, 0.1% Tween-20) supplemented with 50 ng/ml antisense riboprobe. Hybridization was carried out overnight in a humidified chamber at 70 °C. Slides were washed twice with 2x SSC and twice with 0.2x SSC for 30 min each at 68 °C. For probe detection, the sections were incubated with the anti-DIG antibody AP Fragment (1:2000, Sigma-Aldrich) overnight at 4 °C, and after several washes with PBST and NTMT, staining was developed with BM-Purple (Sigma-Aldrich).

For FISH, the anti-digoxigenin-HRP (POD, 1:300, Sigma-Aldrich) antibody was used, and fluorescence detection was performed by tyramide signal amplification for 20 min with a 1:250 dilution of laboratory-synthesized TAMRA or FITC tyramides (70, 71).

### Immunohistochemistry

Immunohistochemistry of zebrafish embryos or brain frozen sections were processed using standard protocols described previously (43, 69).

The following primary and secondary antibodies were used: monoclonal 3F10 rat anti-HA antibody (1:500; Sigma), monoclonal mouse parvalbumin (1:400; Sigma), monoclonal mouse anti-ZebrinII (1:500; a gift from Richard Hawkes, University of Calgary, Canada), Synaptophysin (1:500; Abcam), goat anti-mouse IgG Alexa Fluor-488 (1:1000; Invitrogen/Life Technologies) and goat anti-mouse IgG Alexa Fluor-546 (1:1000; Invitrogen/Life Technologies), goat anti-rat IgG Alexa Fluor 546 (1:1000; Invitrogen/Life Technologies), sheep anti-DIG-AP antibody (1:5000; Sigma-Aldrich), sheep anti-DIG-POD antibody (1:300; Sigma-Aldrich), and sheep anti-FLUO-POD antibody (1:300; Sigma-Aldrich). For detailed information about antibodies that were used please see Table S1.

### Imaging

A laser scanning confocal microscope (TCS SP8, Leica Microsystems) with a 10× air or a 40× water objective was used for fluorescence photo documentation and live imaging. For live imaging, larvae were anesthetized in 0.04 mg/ml tricaine (MS-222, Sigma-Aldrich) and embedded in 1.5% low melting agarose dissolved in 30% Danieau on glass-bottomed imaging dishes. Z-Stack projections and reconstructions were performed with the LAS X software (Leica Microsystems GmbH).

Chromogenic stained sections and larvae were imaged using a Leica MZ FLIII stereomicroscope or a Leitz DM RBE microscope, both equipped with Nikon DS-Vi1 cameras (Nikon). Fluorescence images of frozen sections were either captured on the Leica SP8 or a transmitted light fluorescence microscope equipped with Leica DFC 3000G and Leica DMC 2900 cameras (all Leica Microsystems GmbH).

### Measurements and cell counting

All measurements such as the length or area of PC hemispheres and manual cell counting were performed with Fiji software (http://fiji.sc). All distances were calculated by the use of the Fiji measure tool. The rostral most gap between the PC hemispheres was used to determine the da (Fig. 3*N*, da) and similarly the caudal-most gap was used to define the dp (Fig. 3*N*, dp). For the ap length, the distance from the rostral to the caudal-most part of the PC layer was measured (Fig. 3*N*, ap). The area covered by the PC population in transgenic PC-RFP and PC-Dyrk1A larvae was determined with the Fiji wand tool. The number of PCs was determined by means of the Fiji cell count plugin by stepping through images from z-stacks recorded by confocal microscopy. Quantification of Syn-positive synapses was performed manually with the counter module of Fiji software on magnified confocal images. The area counted in the different sections was kept constant using a rectangle of defined size (40 × 20 μm).

### Dyrk inhibitor treatment

Dyrk inhibitors were dissolved in DMSO before treatment and diluted in 30% Danieau to obtain final concentrations of 2.5 μM for ProINDY, 2.0 μM for Leucettine L41 (Biomol GmbH), both with 0.1% DMSO, and 5 or 10 μM for KuFal194 with 0.2% DMSO. The inhibitors, or Danieau/0.1% DMSO or Danieau/0.2% DMSO, respectively, were then applied to Tg(*ca8*-E1B:FMATagRFP)^bz4^ and Tg(*ca8*-E1b:FynmClover,HA-hDyrk1A)^bz19^ embryos (dechorionated) on 90-mm plates with a maximum of 40 embryos per plate. The treatment was carried out between 3 dpf and 7 dpf with daily replacement of the inhibitor and DMSO solutions. Life imaging of at least eight embryos per treatment group and day was performed between 5 and 7 dpf (2, 3, and 4 dpt).

### Behavioral analysis

Swimming behavior of 5 dpf Tg(*ca8*-E1b:FynmClover,HA-hDyrk1A)^bz19^ and WT zebrafish larvae was recorded using the Viewpoint ZebraBox video tracking system (Viewpoint Life Science, Version 2.5) that is equipped with a high-speed infrared camera. The recording chamber was illuminated with infrared and white lights. Individual larvae were transferred in one well of a 24-well plate, filled with 1 ml 30% Danieau. After habituation to the new environment for 30 min, activity was recorded at 1-min intervals for a total of 30 min. The speed threshold values for slow movements were defined as 0.2< swim speed <21 mm/s and those for fast movements as >21 mm/s. Activity recording data files were exported and further processed in Excel (Microsoft Corp). Recordings were analyzed for total distance traveled, the distance traveled with slow and fast movements, and the speed. The mean swim speed was calculated from the distance covered and the time that the larvae spent swimming during the 30-min recording.

### Statistical analysis

All values illustrated are expressed as the means ± SD. *p* values <0.05 were considered significant. The statistical evaluation was performed by two-way and three-way ANOVA followed by Tukey’s post hoc multiple comparisons test. The two-sided unpaired Student's *t* test was performed for the evaluation of statistical significance between two groups with normal distribution. For non-normally distributed data, the nonparametric *t* test (Mann–Whitney test) was used.

## Ethics statement

All zebrafish experiments were performed according to EU guidelines and were approved by German legislation, that is, the Niedersächsisches Landesamt für Verbraucherschutz und Lebensmittelsicherheit, LAVES (EU Directive 2010_63, license AZ 33.19-42502-04-17/2693).

## Data availability

All data and genetic tools are contained with the article or available on request by contacting the corresponding authors: a.buchberger@tu-braunschweig.de or r.koester@tu-braunschweig.de.

## Supporting information

This article contains supporting information.

## Conflict of interest

The authors declare that they have no conflict of interest with the contents of this article.
