# Supplementary material for: A novel inhibitor rescues cerebellar defects in a zebrafish model of Down syndrome–associated kinase Dyrk1A overexpression
Source: J Biol Chem. 2021 Jun 4;297(1):100853. doi: 10.1016/j.jbc.2021.100853 (PMC8239740; doi:10.1016/j.jbc.2021.100853)
Supplement: Supplemental Figures S1–S4 and Table S1 [file mmc1.pdf]

Supporting Information

Homology modelling

The homology models of zebrafish Dyrk1Aa/Ab (Dyrk1Aa: NP_001074158.1 Lys139-Lys485; Dyrk1Ab: NP_001334760.1 Lys96-Lys442) were built with the tool "homology Model" of molecular operating environment (MOE, version 2018.01) (*Molecular Operating Environment (MOE)*, 2018.01; Chemical Computing Group ULC, 1010 Sherbooke St. West, Suite #910, Montreal, QC, Canada, H3A 2R7, **2018**). First, a homology match search for the query sequence was performed in the Protein Data Base (PBD) (rcsb.org) (Berman et al., 2000). The best matches were examined with respect to their sequence and structure similarity. As the highest similarity was obtained with hDyrk1A (PDB: 4YLK), this was chosen as template for the model in the "homology model" panel. The final model was generated by energy minimization of the best of 10 intermediate models. The intermediate models were ranked with the scoring method GB/VI. The final model was saved as a pdb-file.

Molecular Docking Studies

Molecular docking studies were performed with GOLD (version 5.2.2) (Jones, Willett, Glen, Leach, & Taylor, 1997) on a Windows 7 system. For docking studies with hDyrk1A a new pdb-file was prepared from the crystal structure 4YLJ with UCSF Chimera (version 1.12, Resource for Biocomputing, Visualization, and Informatics at the University of California, San Francisco, USA [supported by NIGMS P41-GM103311]) (Pettersen et al., 2004), where only chain A was present. Protein structures were refined, protonated and energy minimized with the "Quick Prep" function of MOE (version 2018.01) by selecting following options: "use Structure Preparation", "Use Protonate 3D for Protonation" and "Allow ASN/GLN/HIS Flips I Protonate3D", "Fix Atoms Farther than 8 Å from Ligands", "Hydrogens close to Ligands will not be Fixed", "Tether Receptor" (Strength 10, Buffer 0.25), "Delete Water Molecules farther then 4.5 Å from Ligand or Receptor/Ligand" and "Refine" (RMS Gradient of 0.1 kcal/mol/Å). The prepared protein was saved as a mol2-file. The ligands were created with ChemDraw (version 19.1). Protonation and energy minimization was performed with "Protonate 3D" (T = 300, pH = 7.4, Salt = 0.1, Electrostatics: GB/VI, Dielectric: 2, van der Waals: 800R3, Cutoff (A): 15, Solvent: 80, Cutoff (A): 10; enable disconnected metal treatment) and "Energy Minimize"

(Forcefield: Amber 10:EHT, R-Field 1:80, Cutoff (8,10); Cell: No Periodicity; Charges: The System Appears Reasonable; Constraints: Rigid Water Molecules; Gradient: 0.1 RMS kcal/mol/Å²) in MOE and the prepared ligand was saved as mol2-file. The docking was performed with the wizard of GOLD in the HERMES interface (version 1.6.2, CCDC Software Ltd., Cambridge, UK). Missing hydrogen atoms were added, and the ligands and all water molecules distant from the binding pocket were removed from the protein structure. The binding site was defined as a zone of 10 Å around the co-crystallized inhibitor for hDyrk1A or around Lys193 in zebrafish Dyrk1Aa or Lys158 in zebrafish Dyrk1Ab. The implemented scoring function chemscore_kinase was used for evaluation and ranking of the docking results. The search efficiency was set to 200%, the function “generate diverse solution” was activated and the option “allow early termination” was turned off. If water molecules were retained, the option “toggle” was used. For INDY and KuFal194 10 GA runs, for Leucettine L41 20 GA runs were performed. Furthermore, constraints were defined for Leucettine L41: HBond and Distance constraint between benzodioxol-1-oxygen to Leu246 (Dyrk1Aa) or Leu211 (Dyrk1Ab) and Hbond constraint between carbonyl oxygen atom to Lys 193 (Dyrk1Aa) or Lys158 (Dyrk1Ab). Analysis and visualization of the docking results was performed with UCSF ChimeraX (Resource for Biocomputing, Visualization, and Informatics at the University of California, San Francisco, with support from NIH R01-GM129325 and the Office of Cyber Infrastructure and Computational Biology, National Institute of Allergy and Infectious Diseases) (Goddard et al., 2018; Pettersen et al., 2021).

References

Berman, H. M., Westbrook, J., Feng, Z., Gilliland, G., Bhat, T. N., Weissig, H., … Bourne, P. E. (2000). The Protein Data Bank. *Nucleic Acids Research*, *28*(1), 235–242. https://doi.org/10.1093/nar/28.1.235

Goddard, T. D., Huang, C. C., Meng, E. C., Pettersen, E. F., Couch, G. S., Morris, J. H., & Ferrin, T. E. (2018). UCSF ChimeraX: Meeting modern challenges in visualization and analysis. *Protein Science*, *27*(1), 14–25. https://doi.org/https://doi.org/10.1002/pro.3235

Jones, G., Willett, P., Glen, R. C., Leach, A. R., & Taylor, R. (1997). Development and validation of a genetic algorithm for flexible docking11Edited by F. E. Cohen. *Journal of Molecular Biology*, *267*(3), 727–748. https://doi.org/https://doi.org/10.1006/jmbi.1996.0897

Pettersen, E. F., Goddard, T. D., Huang, C. C., Couch, G. S., Greenblatt, D. M., Meng, E. C., & Ferrin, T. E. (2004). UCSF Chimera—A visualization system for exploratory research and analysis. *Journal of Computational Chemistry*, *25*(13), 1605–1612. https://doi.org/https://doi.org/10.1002/jcc.20084

Pettersen, E. F., Goddard, T. D., Huang, C. C., Meng, E. C., Couch, G. S., Croll, T. I., … Ferrin, T. E. (2021). UCSF ChimeraX: Structure visualization for researchers, educators, and developers. *Protein Science*, *30*(1), 70–82. https://doi.org/https://doi.org/10.1002/pro.3943

Figure S1: Specificity of whole mount in situ hybridization probes. A-D RNA in situ hybridization in 5dpf old zebrafish larvae with *dyrk1aa* specific antisense (**A**) and sense as well as (**B**) and *dyrk1ab* specific antisense (**C**) and sense (**D**) probes. While sense generate no specific staining pattern, both antisense probes reveal widespread expression of *dyrk1aa* and *dyrk1ab* throughout the nervous system. Scale bar: 200 µm.

Figure S2: Similar binding mode for DYRK1a inhibitors predicted by docking studies on zebrafish Dyrk1Aa/Dyrk1Ab homology models. A-C Overall structures of hDyrk1A (PDB:4YLJ) (**A**), zebrafish Dyrk1Aa (**B**) and zebrafish Dyrk1Ab (**C**) for homologous regions. **D-F** Comparison of the binding mode of the different Dyrk1a inhibitors in the hDyrk1A (yellow, PDB: 4YLJ), the homology model of zebrafish Dyrk1Aa (red) and Dyrk1Ab (blue). An overlay of the respective surface structures of the different ATP-binding pockets is shown, in which red and blue colors indicate deviations of the zebrafish homology models from the human crystal structure. The color of the inhibitor indicates its position in the corresponding structure. The individual binding modes of the inhibitors in hDyrk1a were obtained from the co-crystal structures of (**D**) INDY (PDB 4ANQ), (**E**) KuFal194 (PDB: 4YLJ) and (**F**) Leucettine L41 (PDB 4AZE). For the inhibitor binding modes in zebrafish Dyrk1Aa/Ab docking studies were performed. Note: Instead of ProINDY, the active form INDY was used, which is formed *in vivo* by deacetylation. While INDY and KuFal194 adapt a similar binding mode in human and zebrafish Dyrk1a homologs, the use of constraints is necessary to obtain a similar binding mode for Leucettine L41 (see Figure S3).

Figure S3: Analysis of molecular docking studies for Leucettine L41 in human Dyrk1A and zebrafish homology models. A-B An overlay of the respective surface

structures of the different ATP-binding pockets is shown, in which red and blue colors indicate deviations of the zebrafish homology models from the human crystal structure. The color of the inhibitor indicates its position in the corresponding structure. **A** Overlay of Leucettine L41 binding modes from hDyrk1a (yellow, PDB: 4AZE) and the top ranked docking results in zebrafish homology model Dyrk1Aa (red) and Dyrk1Ab (blue). The docking was performed without using constraints. **B** Analogous overlay of Leucettine L41 binding modes in the presence of one distance constraint and two hydrogen bonds. **C** Visualisation of the Leucettine L41 docking result in hDyrk1a (PDB: 4YLJ). The actual binding mode from the crystal structure (PDB: 4AZE) is shown in yellow, and the predicted binding mode is shown in red. Even without using constraints, the two binding modes show good superposition with slightly different orientation of the aniline substituent. **D** Illustration of the constraint anchor points on Leucettine L41. Constraints were defined for the benzodioxol-oxygen to the backbone NH of Leu in the hinge region (Leu246 in Dyrk1Aa and Leu211 in Dyrk1Ab, hydrogen bond and distance) and the carbonyl oxygen atom to Lys in the back of the ATP binding pocket (Lys193 in Dyrk1Aa and Lys158 in Dyrk1Ab, hydrogen bond).

Figure S4: The Dryk1A inhibitor KuFal194 rescues the morphological Purkinje cell layer impairments of PC-Dyrk1A larvae. KuFal194 dissolved in DMSO was diluted to 5 µM in 30% Danieau. 3dpf larvae were treated for 4 days and the inhibitor was changed daily. *In vivo* imaging of PC hemispheres of all groups was performed between 2 and 4 dpt. Measurements of the anterior distance (µm) of the PC hemispheres were performed as shown in Figure 7. The following groups were tested: PC-RFP control larvae treated with DMSO (Ctrl, DMSO), PC-RFP control larvae treated with 5 µM KuFal194 (Ctrl + KuFal), PC-Dyrk1A larvae treated with DMSO (Dyrk1A DMSO) and PC-Dyrk1A larvae treated with 5 µM KuFal194 (Dyrk1A + KuFal). Data are mean ± S.D. (error bars). Statistical analysis was performed using three-way ANOVA, followed by Tukey's post-hoc multiple comparisons test. Row factor: $F = 1.389$, $p=0.2519$; row factor (Ctrl. DMSO Ctrl. KuFal194 vs. Dyrk1A DMSO Dyrk1A KuFal194): $F = 161.8$, $p<0.0001$; row factor (Ctrl. DMSO Dyrk1A DMSO vs. Ctrl KuFal194 Dyrk1A KuFal194): $F = 11.48$, $p=0.0009$; row factor x (Ctrl. DMSO Ctrl. KuFal194 vs. Dyrk1A DMSO Dyrk1A KuFal194) $F = 5.579$, $p=0.0044$; row factor x (Ctrl. DMSO Dyrk1A DMSO vs. Ctrl KuFal194 Dyrk1A KuFal194): $F = 0.4429$, $p=0.6429$; row factor (Ctrl. DMSO Ctrl. KuFal194 vs. Dyrk1A DMSO Dyrk1A KuFal194) x (Ctrl. DMSO

Dyrk1A DMSO vs. Ctrl KuFal194 Dyrk1A KuFal194): F = 12.06, p<0.0006; row factor x (Ctrl. DMSO Ctrl. KuFal194 vs. Dyrk1A DMSO Dyrk1A KuFal194) x (Ctrl. DMSO Dyrk1A DMSO vs. Ctrl KuFal194 Dyrk1A KuFal194): F= 1.580, p=0.2088. *Ctrl. DMSO vs. Dyrk1A DMSO 2dpt p=0.0105; *Ctrl DMSO vs Dyrk1A DMSO 3dpt p>0.0001; Ctrl DMSO vs. Dyrk1A DMSO 4dpt p>0.0001; Dyrk1A DMSO vs. Dyrk1A KuFal 5µM 4dpt p>0.0001. *p=0.0105; ****p<0.0001; ns: not significant.

Table S1: List of antibodies

**Primary Antibody**	**Company**	**Catalog Number**	**Dilution**	**Species/subtype**
Anti-HA	Sigma	11867423001	1:500	Rat IgG_1
Anti-Parvalbumin				
Anti-Synaptophysin	Abcam	Ab32594	1:500	Rabbit, polyclonal, IgG
Anti-ZebrinII	Provided by Richard Hawkes		1:500	Mouse, monoclonal, IgG
Anti-digoxigenin AP, Fab fragments	Sigma	11093274901	1:5000	Sheep, polyclonal IgG
Anti DIG-POD	Sigma	11207733910	1:300	Sheep, polyclonal
Anti-Fluorescein-POD	Sigma	11426346910	1:300	Sheep, polyclonal
**Secondary Antibody**	**Company**	**Catalog Number**	**Dilution**	**Fluorophore**
Goat Alexa anti-mouse IgG	ThermoFisher/Invitrogen	A11001	1:1000	Alexa488
Goat Alexa anti-mouse IgG	ThermoFisher/Invitrogen	A11030	1:1000	Alexa546
Goat Alexa anti-mouse IgG	ThermoFisher/Invitrogen	A11031	1:1000	Alexa568
Goat Alexa anti-rat IgG	ThermoFisher/Invitrogen	A11077	1:1000	Alexa568
Goat Alexa anti-rabbit IgG	ThermoFisher/Invitrogen	A11079	1:000	Alexa568

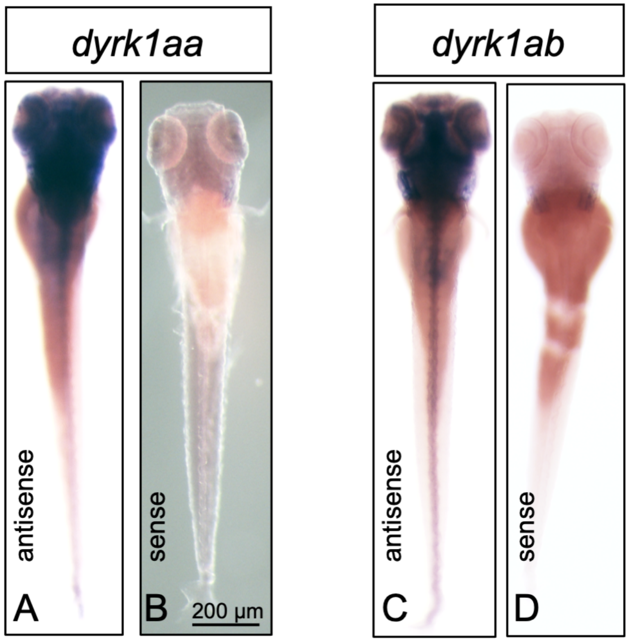
dyrk1aa
dyrk1ab
antisense
sense
antisense
sense
A
B
200 µm
C
D

Figure S1

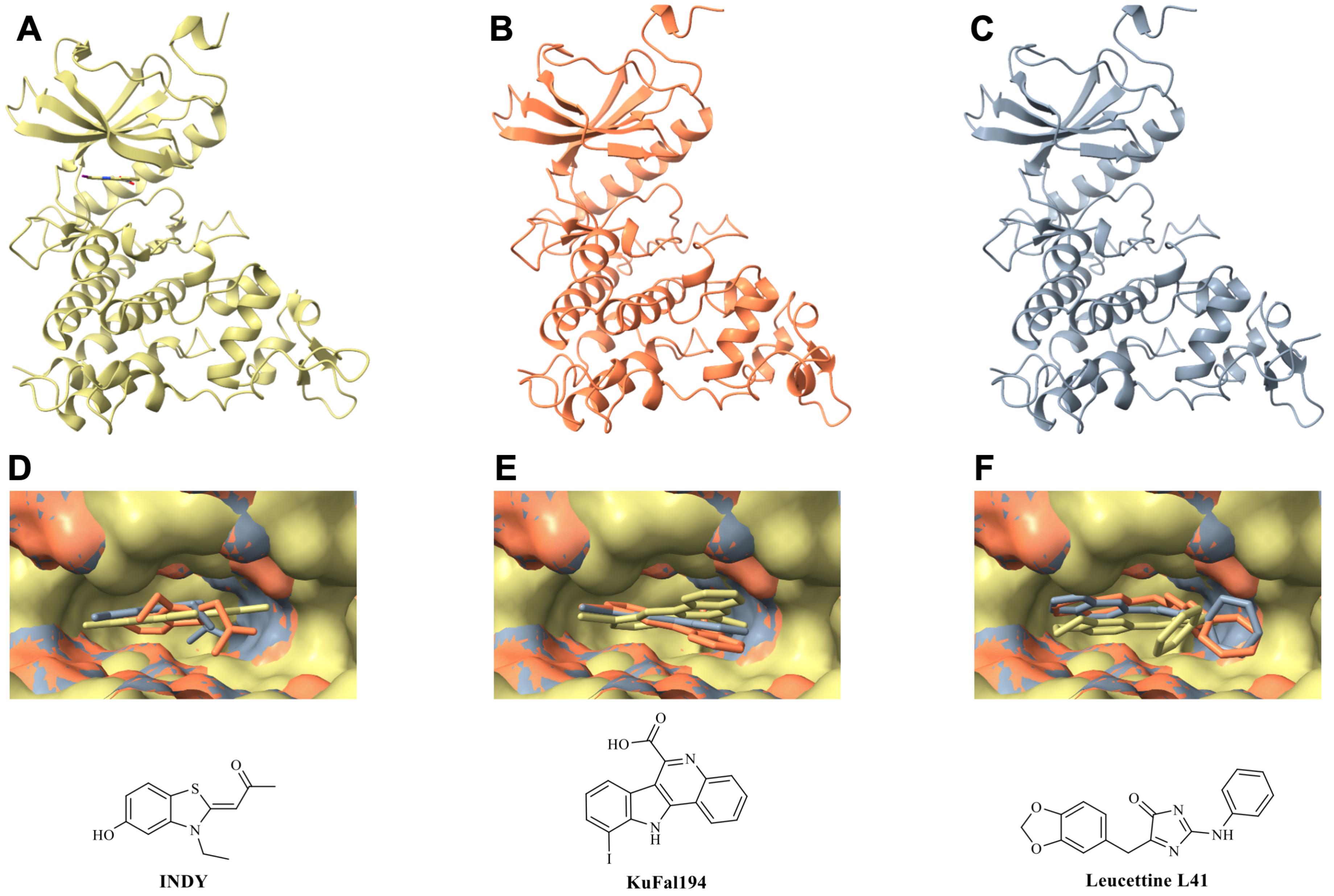
A
B
C
D
E
F
HO
S
N
O
INDY
HO
O
N
N
H
I
KuFal194
O
O
O
N
N
NH
Leucettine L41

Figure S2

A **without constraints**

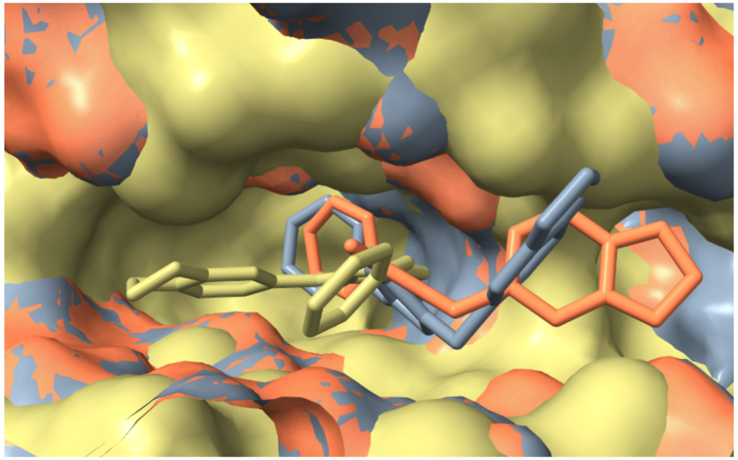

B **Constraints** (2x Hbonds, 1x Distance)

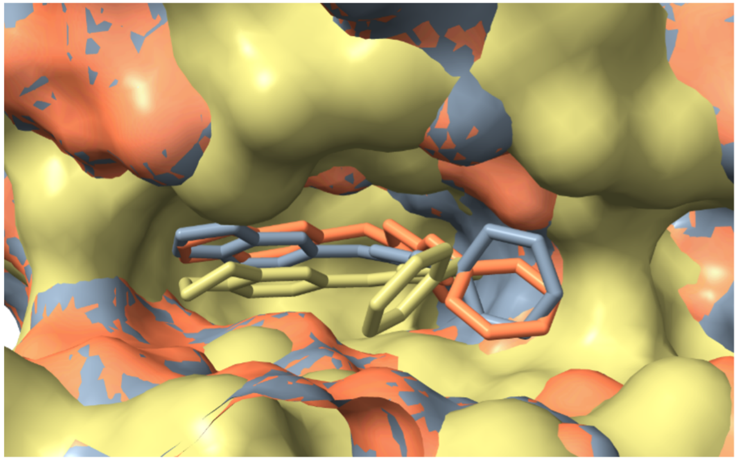

C **Docking in hDYRK1A without constraints**

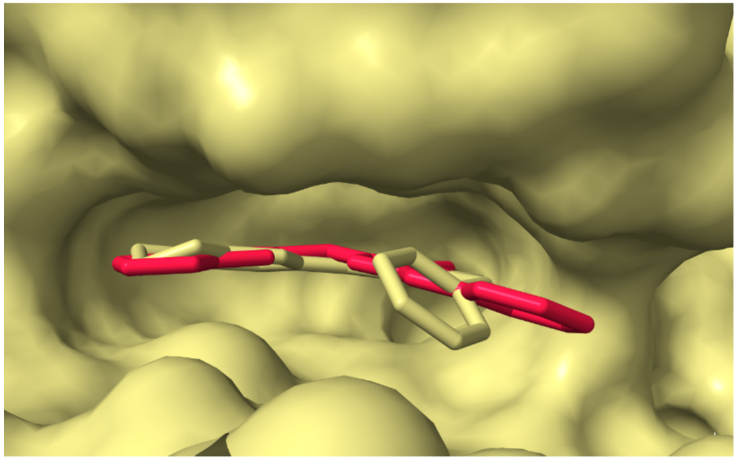

D

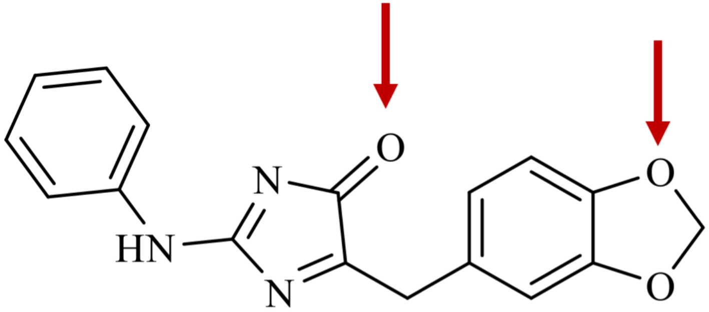

Leucettine L41

Figure S3

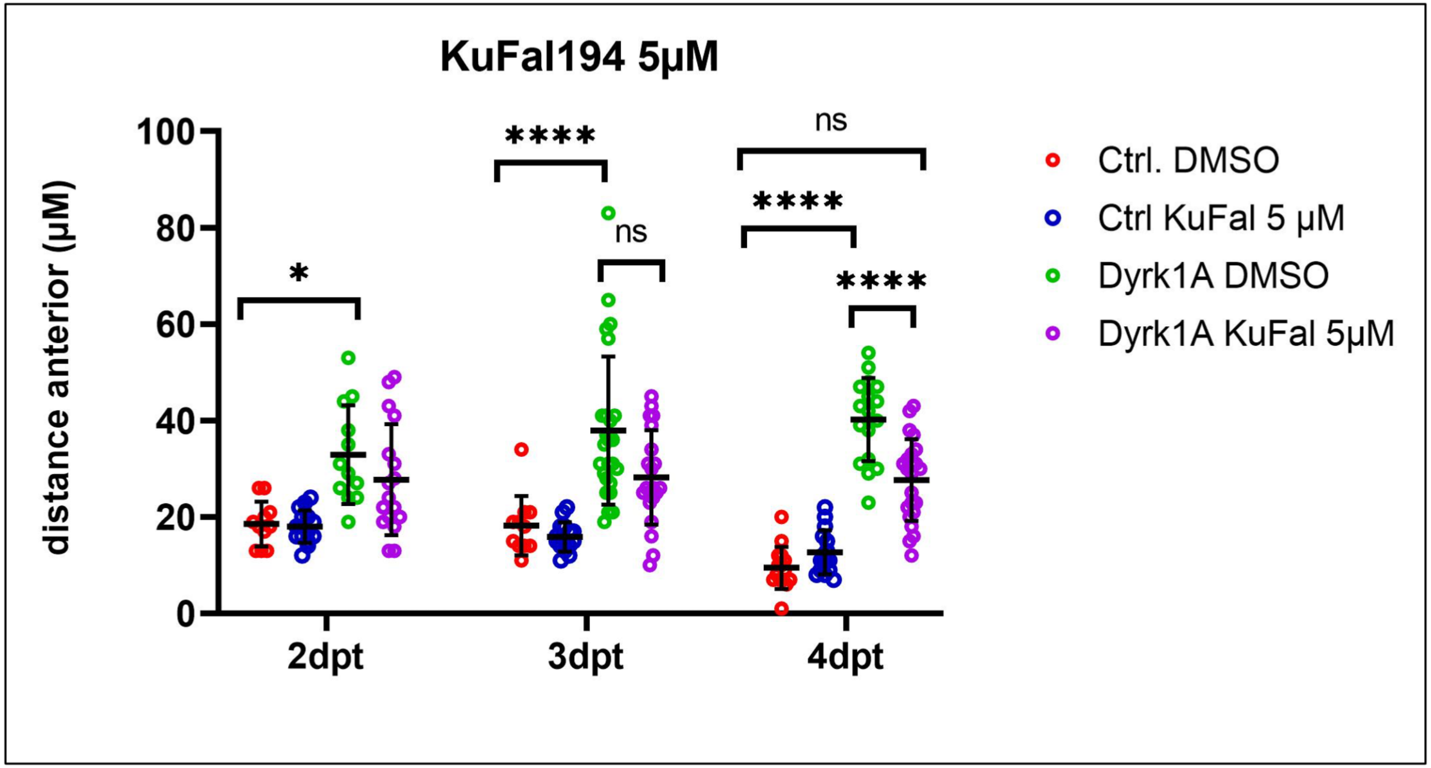
KuFal194 5µM
distance anterior (µM)
100
80
60
40
20
0
2dpt
3dpt
4dpt
*
****
ns
****
****
ns
Ctrl. DMSO
Ctrl KuFal 5 µM
Dyrk1A DMSO
Dyrk1A KuFal 5µM

Figure S4
